# Effect of physical activity on pulmonary function and quality of life in asthma patients: a systematic review and meta-analysis

**DOI:** 10.3389/fmed.2026.1848571

**Published:** 2026-06-12

**Authors:** Zhicheng Zhu, Youjia Mao, Yong Fan, Zijian Zhu, Lisha Xie, Cui Huang

**Affiliations:** School of Physical Education, Xinyu University, Xinyu, Jiangxi, China

**Keywords:** asthma, exercise, FEV_1_, meta-analysis, physical activity, pulmonary function, quality of life, systematic review

## Abstract

**Background:**

Physical activity is increasingly recognized as a non-pharmacological adjunct in asthma management, yet existing systematic reviews are limited by single-modality scope, narrow age ranges, and inconsistent operationalization of pulmonary function outcomes. We comprehensively evaluated the effects of physical activity on pulmonary function and quality of life in patients with asthma across a broad age range and multiple exercise modalities.

**Methods:**

We systematically searched PubMed, Embase, Cochrane Library, Web of Science, MEDLINE, and CNKI up to April 25, 2026, for randomized controlled trials (RCTs) of structured physical activity interventions ( ≥ 6 weeks) in asthma. The primary outcome was forced expiratory volume in 1 s as a percentage of predicted value (FEV_1_% predicted); secondary outcomes were FVC% predicted, PEF% predicted, PEF (L/s), FEV_1_ (L), FVC (L), and the Pediatric Asthma Quality of Life Questionnaire (PAQLQ). Random-effects meta-analyses used the restricted maximum likelihood estimator. Heterogeneity was explored via subgroup analyses and meta-regression. Risk of bias was assessed with Cochrane RoB 2 and certainty of evidence with GRADE.

**Results:**

Twenty-two RCTs (*n* = 1,280) were included. Physical activity significantly improved FEV_1_% predicted (MD = 5.79, 95% CI: 1.89–9.69; *I*^2^ = 89.16%), PEF (L/s) (MD = 0.47, 95% CI: 0.08–0.85), FVC (L) (MD = 0.55, 95% CI: 0.25–0.84), and PAQLQ (MD = 1.13, 95% CI: 0.45–1.81), but not FVC% predicted, PEF% predicted, or FEV_1_ (L). Meta-regression did not identify intervention duration, weekly frequency, session duration, or intervention type as significant moderators for either FEV_1_% predicted or FVC% predicted (all *P* > 0.20). Egger’s test and trim-and-fill indicated no significant publication bias. Certainty of evidence was low for FEV_1_% predicted and very low for FVC% predicted, PEF% predicted, and PAQLQ.

**Conclusion:**

Physical activity is associated with improved FEV_1_% predicted and PAQLQ in patients with asthma, but overall certainty is low, mainly due to risk of bias and heterogeneity. The systematic discordance between predicted-value and absolute-unit scales across pulmonary function outcomes highlights the need for standardized outcome reporting in future trials.

## 1 Introduction

Asthma is the most prevalent chronic respiratory disease, commonly characterized by recurrent symptoms such as chest tightness, coughing, shortness of breath, and wheezing. Its primary features include persistent airway inflammation, airway hyperresponsiveness, bronchoconstriction, and excessive mucus secretion; in severe cases, asthma can be life-threatening (1). According to the Global Asthma Report, the global prevalence of asthma is 9.1% among children, 11.0% among adolescents, and 6.6% among adults (2). Compared with their healthy peers, adolescents with asthma are more susceptible to emotional dysregulation and experience a marked decline in health-related quality of life (3), which in turn increases the risk of acute exacerbations and mortality. Anxiety and comorbid depression in caregivers may further reduce treatment adherence, compromising symptom control and clinical outcomes (4). Among adults, asthma heterogeneity and varying degrees of disease control—particularly in uncontrolled cases—lead to multidimensional impairments across physical, psychological, and social health domains (5, 6).

Evidence-based research indicates that asthma, a chronic inflammatory airway disease, currently lacks a curative pharmacological treatment (7). The cornerstone of pharmacotherapy is inhaled corticosteroids (ICS) combined with long-acting β_2_-agonists (8). Although this approach is effective and well-established, it remains inadequate for symptom control in patients with severe asthma (9, 10), and long-term use is associated with serious adverse effects (11). Moreover, adult patients with asthma frequently demonstrate suboptimal medication adherence due to forgetfulness, cognitive biases concerning adverse drug reactions, and the financial burden of treatment (12). Against this backdrop, physical activity has emerged as a promising non-pharmacological intervention. By tailoring training parameters to individual tolerance thresholds, psychological readiness, and perceived exertion, physical activity offers substantial advantages in enhancing treatment adherence and cross-age applicability among patients with asthma (13–15).

A clear conceptual distinction is warranted at the outset between physical activity and physical exercise. The World Health Organization (WHO) defines physical activity as any bodily movement produced by skeletal muscles that requires energy expenditure, referring to all movement during leisure time, for transport, or as part of a person’s work or domestic activities (16). WHO further notes that physical activity should not be confused with exercise, which is a planned, structured, and repetitive subcategory of physical activity that aims to improve or maintain one or more components of physical fitness (16). The trials synthesized in the present review involve structured interventions that include both walking and pulmonary rehabilitation programs (which are typically classified as physical activity) and modalities such as swimming, aerobic training on a bike or treadmill, cycling, and high-intensity interval training (which are more accurately described as physical exercise). For clarity and consistency throughout this manuscript, we use the umbrella term physical activity to refer to the full set of interventions evaluated, while preserving the specific modality names (e.g., aerobic exercise, yoga, breathing exercises) when describing individual trials (17, 18).

Although physical activity has shown promise as a non-pharmacological intervention for asthma, the evidence base remains incomplete and methodologically inconsistent. First, the clinical safety of physical activity in asthma is not universally established: some studies suggest that an abrupt increase in airflow during exercise may transiently narrow the airways and potentially trigger bronchospasm (19, 20). Second, existing systematic reviews and meta-analyses face important methodological limitations. Most previous reviews have been restricted to a single exercise modality—aerobic exercise (21), swimming (22), yoga (23, 24), or high-intensity interval training (25)—or to a specific age group, with separate syntheses for children (26, 27) and adults (21, 28), thereby limiting the breadth of available evidence. Third, an underappreciated methodological challenge concerns the operationalization of pulmonary function outcomes: studies inconsistently report outcomes as absolute values (e.g., FEV_1_ in liters) or as percentages of predicted values (% predicted) derived from different reference equations (29, 30). Absolute values are confounded by anthropometric variables such as height, age, sex, and ethnicity, compromising cross-study comparability; yet when both formats are pooled together, the standardized mean difference (SMD) must be used, further diminishing clinical interpretability (24, 28) Finally (28), few reviews have systematically examined potential moderating factors—such as exercise type, intervention duration, frequency, and session length—that are essential for translating aggregate evidence into actionable clinical recommendations.

To address these gaps, the present systematic review and meta-analysis aims to comprehensively evaluate the effects of physical activity on pulmonary function (FEV_1_% predicted, FVC% predicted, PEF% predicted, and the corresponding absolute-unit scales) and quality of life (PAQLQ) in patients with asthma across a broad age range, incorporating multiple exercise modalities and systematically investigating moderating intervention parameters through subgroup analyses and meta-regression.

## 2 Methods

This systematic review was registered in the International Prospective Register of Systematic Reviews (PROSPERO; registration ID: CRD420251026001) and was conducted in accordance with the Preferred Reporting Items for Systematic Reviews and Meta-Analyses (PRISMA) 2020 statement. The processes of study selection, eligibility assessment, data extraction, and statistical analysis followed a predefined protocol based on the Cochrane Collaboration guidelines.

### 2.1 Eligibility criteria

Eligibility criteria for inclusion were specified in advance using the PICOS framework:

(1) Population (P): Patients with physician-diagnosed asthma, with no restriction on age, sex, ethnicity, or asthma severity.

(2) Intervention (I): Any structured physical activity intervention, including but not limited to aerobic exercise (e.g., swimming, jogging, cycling), resistance training, breathing exercises, yoga, or combinations of multiple modalities, with a minimum duration of 6 weeks.

(3) Comparator (C): Usual care, no intervention, waiting-list control, non-exercise control, or pharmacological treatment with low-dose ICS and the short-acting β_2_-agonist salbutamol.

(4) Outcomes (O): Studies reporting at least one of the following: forced expiratory volume in 1 s (FEV_1_; in liters or as a percentage of predicted value), forced vital capacity (FVC; in liters or as a percentage of predicted value), peak expiratory flow (PEF; as a percentage of predicted value or in L/s), or the Pediatric Asthma Quality of Life Questionnaire (PAQLQ) total score.

(5) Study design (S): Randomized controlled trials (RCTs), including parallel-group and cluster-randomized designs.

Studies were excluded if they met any of the following criteria: (1) non-randomized studies, including quasi-experimental studies, observational studies, case reports, case series, reviews, editorials, conference abstracts without full text, and animal studies; (2) participants with concurrent respiratory or systemic diseases (e.g., chronic obstructive pulmonary disease, cystic fibrosis, bronchiectasis, severe cardiovascular disease) that could confound the assessment of asthma-specific outcomes; (3) interventions that did not include a structured physical activity component, such as pharmacological-only interventions, dietary interventions, or educational programs without an exercise component; (4) intervention duration shorter than 6 weeks, as such durations are typically insufficient to elicit measurable physiological adaptations; (5) duplicate publications or studies with overlapping data, in which case the version with the most complete data was retained.

#### 2.1.1 Grouping of studies for synthesis

For subgroup analyses, included studies were grouped a priori according to the following variables: (1) participant age ( < 18 years vs. ≥ 18 years); (2) intervention type (aerobic vs. non-aerobic); (3) total intervention duration ( > 8 weeks vs. ≤ 8 weeks); (4) weekly frequency ( > 3 sessions/week vs. ≤ 3 sessions/week); and (5) session duration ( ≥ 45 min vs. < 45 min).

### 2.2 Information sources

We systematically searched six electronic databases: PubMed, Embase, Cochrane Library, Web of Science, MEDLINE, and the China National Knowledge Infrastructure (CNKI). The initial search across all databases was conducted on April 4, 2025. To incorporate recently published studies, an updated search was performed on April 25, 2026; because the proportion of Chinese-language studies meeting our inclusion criteria was low during the initial search, CNKI was not re-searched in the update. No language or publication date restrictions were applied. We additionally hand-searched the reference lists of the included studies to identify potentially eligible studies that may have been missed.

### 2.3 Search strategy

The search strategy was constructed using the Cochrane Highly Sensitive Search Strategy (CHSSS) framework, incorporating all Medical Subject Headings (MeSH) and free-text terms related to “physical activity” and “asthma.” Search terms were combined using Boolean operators (AND/OR/NOT) and adapted to the syntax of each database. The complete search strategy for each database, including the MeSH terms, free-text terms, filters, and combinations used, is provided in Supplementary Table 1.

### 2.4 Selection process

All retrieved records were imported into EndNote 21 for deduplication and reference management. Two reviewers (YJ M and Y F) independently performed the screening: in the first stage, records were screened by title and abstract to exclude clearly ineligible studies, and in the second stage, potentially eligible studies underwent full-text review to confirm eligibility. The judgments of the two reviewers were compared at each stage; disagreements were resolved through discussion, and a third reviewer (ZC Z) was consulted when consensus could not be reached. Neither automated screening tools nor machine learning aids were used. During screening, two reports were identified as different publications of the same underlying study and were merged into a single study record, following the Cochrane Handbook recommendations.

### 2.5 Data collection process

Two reviewers (YJ M and ZJ Z) independently extracted data from the included studies using a standardized data extraction form predefined in the study protocol. The extracted data were compared; disagreements were resolved through discussion, and a third reviewer (ZC Z) was consulted when consensus could not be reached.

#### 2.5.1 Data acquisition and handling of missing values

When studies reported incomplete data, we first attempted to derive the required statistics from the available information using the standard formulas recommended in the Cochrane Handbook (section 6.5), for example, deriving the standard deviation (SD) from the standard error (SE), deriving the SD from the 95% confidence interval (CI), or estimating the mean and SD from the median and interquartile range. When the conversion was not possible or the required information was entirely missing, the study was excluded from the corresponding pooled analysis but retained for analyses of other outcomes for which data were available.

### 2.6 Data items

#### 2.6.1 Outcome data

For each included study, we collected all result data compatible with the outcomes of this review, including FEV_1_ (in L or % predicted), FVC (in L or % predicted), PEF (in % predicted or L/s), and the PAQLQ total score. When a study reported outcomes at multiple time points, we prioritized the measurement obtained immediately after the end of the intervention.

#### 2.6.2 Other variables

The following variables were extracted from each study: (1) study characteristics: first author, year of publication, country, study design, and sample size; (2) participant characteristics: mean age, sex distribution, asthma severity, and baseline body mass index (BMI); (3) intervention details: type, weekly frequency, session duration, and total intervention duration; (4) comparator details: type of control (usual care, no intervention, waiting-list, etc.).

### 2.7 Study risk of bias assessment

Two reviewers (YJ M and LS X) independently assessed the methodological quality of the included studies using the revised Cochrane Risk-of-Bias 2 tool (RoB 2) for randomized trials (31).

The RoB 2 tool covers five domains: (1) bias arising from the randomization process; (2) bias due to deviations from the intended interventions; (3) bias due to missing outcome data; (4) bias in measurement of the outcome; (5) bias in selection of the reported result. Each domain was judged as “low risk of bias,” “some concerns,” or “high risk of bias” based on the responses to the signaling questions. The overall risk-of-bias judgment was based on the assessments across all domains: studies judged as low risk in all domains were rated as low overall; studies judged as high risk in at least one domain, or as having some concerns in multiple domains in a way that substantially reduced confidence in the result, were rated as high overall; all other studies were rated as having some concerns.

The judgments of the two reviewers were compared and disagreements were resolved through discussion; a third reviewer (ZC Z) was consulted when consensus could not be reached.

### 2.8 Effect measures

All outcomes were continuous variables. We used the mean difference (MD) with the corresponding 95% CI as the effect measure. When the same outcome was reported on different scales across studies (e.g., FEV_1_ reported both in L and as a percentage of predicted value), each scale was pooled separately; no cross-scale standardized pooling (e.g., standardized mean difference, SMD) was performed, in order to preserve the clinical interpretability of the pooled estimates.

### 2.9 Synthesis methods

#### 2.9.1 Determining the eligibility of studies for each synthesis

For each outcome, we tabulated the intervention characteristics, comparator settings, and outcome measurement methods of the included studies to confirm whether they met the predefined criteria for synthesis. Specifically, only studies in which the intervention was a structured physical activity program ( ≥ 6 weeks), the comparator was usual care or no intervention, and the outcome was reported on a compatible scale were included in the synthesis of the corresponding outcome.

#### 2.9.2 Data preparation

When a study did not directly report the SD but provided a 95% CI, we derived the SD from the CI using the method recommended in the Cochrane Handbook (section 6.5.2):


$$\mathrm{SD}=\sqrt{n}\times \left[\left({\mathrm{CI}}_{\mathrm{upper}}-{\mathrm{CI}}_{\mathrm{lower}}\right)/\left(2\times t\,\_\,\left\{\alpha /2,n-1\right\}\right)\right]$$

where *n* is the sample size, α = 0.05, and *t* is the value from the *t*-distribution with the corresponding degrees of freedom. The conversion was performed in Microsoft Excel. When a study reported neither the 95% CI nor any other statistic from which the SD could be derived, the study was excluded from the corresponding pooled analysis. For studies that compared multiple intervention arms with a single shared control group, the control group sample size was proportionally divided across the intervention arms to create independent comparison units, following the Cochrane Handbook (section 6.5.2.10) recommendation, in order to avoid unit-of-analysis errors arising from double-counting of the control group.

#### 2.9.3 Tabulation and graphical display

Characteristics of individual studies are presented in a summary table that includes sample size, participant characteristics, intervention and comparator details, and outcome measurement methods. Pooled results for each outcome are presented as forest plots showing the effect estimate, 95% CI, and weight for each study, together with the pooled effect estimate. For studies with multiple intervention arms, forest plots present each “study–comparison unit” separately so that the source of each effect estimate is identifiable.

#### 2.9.4 Synthesis approach

All meta-analyses were performed using Stata 18.0 software, with statistical significance set at a two-sided *P* < 0.05.

Given the anticipated clinical and methodological heterogeneity across the included studies in participant characteristics, intervention protocols, and outcome measurement, all primary analyses used a random-effects model with the restricted maximum likelihood (REML) estimator for the between-study variance (τ^2^). The original protocol specified the DerSimonian-Laird (DL) estimator; however, based on recent methodological recommendations, we adopted REML as the primary estimator because it provides more accurate estimates of between-study variance under conditions of high heterogeneity or a small number of studies (32).

Statistical heterogeneity was assessed using the *I*^2^ statistic and Cochran’s Q test. *I*^2^ values of 25, 50, and 75% were considered to indicate low, moderate, and high heterogeneity, respectively (33). For outcomes with substantial statistical heterogeneity (*I*^2^ > 50%) and ≥ 10 included studies, we explored heterogeneity sources through subgroup analyses, meta-regression, and sensitivity analyses.

#### 2.9.5 Investigation of heterogeneity

##### 2.9.5.1 Subgroup analyses

For the primary outcome FEV_1_% predicted and the secondary outcome FVC% predicted (both with k ≥ 10 and substantial heterogeneity), subgroup analyses were conducted according to the five predefined grouping variables (see section 2.1). Between-subgroup differences were evaluated using Cochran’s Q test, with *P* < 0.10 considered indicative of a significant subgroup difference (a more lenient threshold was adopted because subgroup comparisons typically have low statistical power). Other secondary outcomes had *k* < 10 and were not subjected to subgroup analyses.

##### 2.9.5.2 Meta-regression

For outcomes with k ≥ 10, random-effects univariable meta-regression was performed to quantitatively assess the moderating effect of continuous covariates on the effect size. The predefined covariates were total intervention duration (weeks), weekly frequency (sessions/week), session duration (minutes), and intervention type (aerobic vs. non-aerobic).

#### 2.9.6 Sensitivity analyses

To assess the robustness of the pooled estimates, we performed the following sensitivity analyses:

(1) Leave-one-out analysis: Each study was omitted in turn and the pooled estimate was recalculated to assess the influence of individual studies on the pooled effect and heterogeneity, and to identify potential outliers or major sources of heterogeneity.

(2) Comparison of heterogeneity variance estimators: In addition to the REML estimator used in the primary analysis, we re-pooled the data using the DL and Paule-Mandel (PM) estimators to verify the robustness of the pooled estimates to the choice of between-study variance estimator.

(3) Outlier removal analysis: Studies identified as major sources of heterogeneity in the leave-one-out analysis were excluded, and the data were re-pooled to assess the effect on the overall estimate and heterogeneity.

### 2.10 Reporting bias assessment

Reporting bias was assessed for outcomes with ≥ 10 included studies. The methods used were: (1) visual inspection of the funnel plot for asymmetry, and (2) Egger’s regression test (34), with *P* < 0.05 considered indicative of significant asymmetry. In addition, the Duval and Tweedie trim-and-fill method (35) was applied to estimate an adjusted pooled effect after imputing potentially missing studies, and the adjusted estimate was compared with the original estimate to evaluate the robustness of the overall conclusion to potential publication bias. Outcomes with fewer than 10 studies were not subjected to formal reporting bias assessment due to insufficient power.

### 2.11 Certainty assessment

We assessed the certainty of evidence for the primary outcome (FEV_1_% predicted) and key secondary outcomes (FVC% predicted, PEF% predicted, and PAQLQ) using the Grading of Recommendations Assessment, Development and Evaluation (GRADE) framework (36). Two assessors (YJ M and ZJ Z) independently evaluated each outcome across the five downgrading factors—(1) risk of bias, (2) inconsistency, (3) indirectness, (4) imprecision, and (5) publication bias—and the three potential upgrading factors—(1) large magnitude of effect, (2) dose–response gradient, and (3) all plausible confounders would reduce the demonstrated effect. The overall certainty of evidence for each outcome was rated as high, moderate, low, or very low. The judgments of the two assessors were compared and disagreements were resolved through discussion; a third assessor (ZC Z) was consulted when needed.

## 3 Results

### 3.1 Study selection

The initial search (April 4, 2025) identified 3,443 records across the six databases (PubMed *n* = 248, Embase *n* = 909, Cochrane Library *n* = 996, MEDLINE *n* = 182, Web of Science *n* = 1,024, CNKI *n* = 84). After removal of 994 duplicates, 2,449 records were screened by title and abstract, and 2,222 irrelevant records were excluded. The remaining 227 reports underwent full-text review, of which 208 were excluded for the following reasons: data not retrievable (*n* = 38), conference abstracts (*n* = 40), did not meet inclusion criteria (*n* = 95), and incompatible outcome measures (*n* = 35). Two reports were identified as duplicate publications of the same study in different journals and were merged into a single study record (37, 38); the initial search ultimately yielded 18 studies (19 reports) (15, 38–54).

**FIGURE 1:**
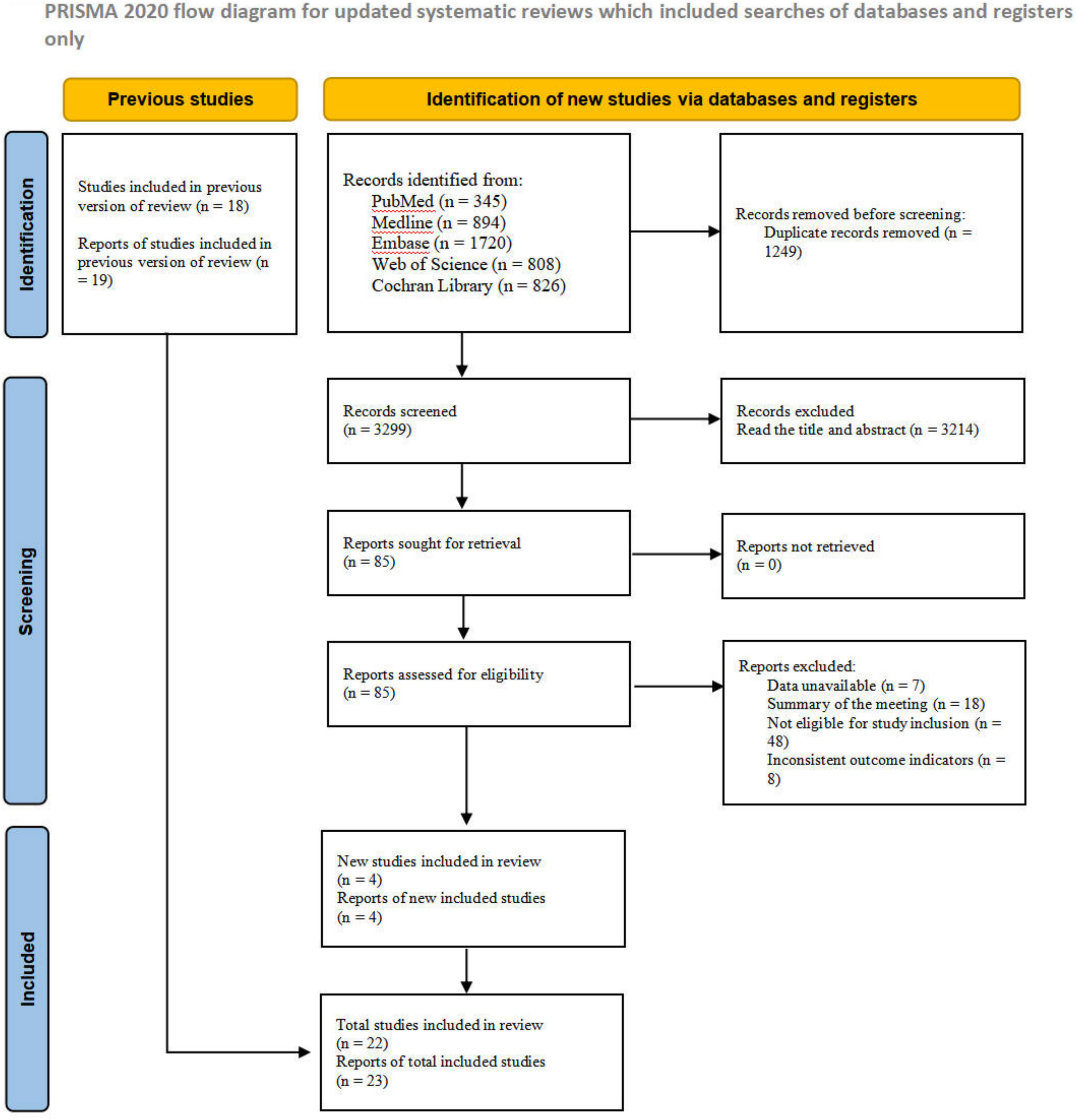
PRISMA 2020 flow diagram of the study selection process. The flow diagram illustrates the identification, screening, eligibility assessment, and inclusion of studies retrieved from the initial search (April 4, 2025) and the updated search (April 25, 2026) across six electronic databases (PubMed, Embase, Cochrane Library, Web of Science, MEDLINE, and CNKI). PRISMA, Preferred Reporting Items for Systematic Reviews and Meta-Analyses; CNKI, China National Knowledge Infrastructure.

The updated search (April 25, 2026) used an expanded search strategy across five databases (CNKI was not re-searched because the yield of Chinese-language studies meeting the inclusion criteria during the initial search was low), identifying 4,593 records (PubMed *n* = 345, MEDLINE *n* = 894, Embase *n* = 1,720, Web of Science *n* = 808, Cochrane Library *n* = 826). After removal of 1,249 duplicates, 3,299 records were screened by title and abstract, and 3,214 irrelevant records were excluded. The remaining 85 reports underwent full-text review, of which 81 were excluded (data not retrievable *n* = 7, conference abstracts *n* = 18, did not meet inclusion criteria *n* = 48, and incompatible outcome measures *n* = 8). The updated search added 4 studies (4 reports) (Figure 1).

Combining both searches, this systematic review included a total of 22 studies (23 reports) (15, 38–58). Pooled analyses were based on the following number of studies and effect sizes: FEV_1_% predicted (primary outcome), 13 studies/16 effect sizes; FVC% predicted, 11 studies/14 effect sizes; PEF% predicted, 7 studies/10 effect sizes; PEF (L/s), 5 studies/7 effect sizes; FEV_1_ (L), 8 studies/10 effect sizes; FVC (L), 6 studies/8 effect sizes; and PAQLQ, 6 studies/6 effect sizes.

During full-text review, the study by Soliman et al. (59) was initially identified as a potentially eligible RCT, but full-text review revealed that the participants were children with asthma comorbid with cerebral palsy (59). The study was therefore excluded per the predefined exclusion criteria.

### 3.2 Study characteristics

This systematic review ultimately included 22 RCTs (23 reports) (15, 38–58), with a total analytic sample of 1,280 patients with asthma. Detailed characteristics of each study are provided in Supplementary Table 2.

#### 3.2.1 Participant characteristics

Sample sizes ranged from 14 (47) to 140 participants (41). The mean age spanned a wide range from children to middle-aged adults: 12 studies included children or adolescents (< 18 years) (39–42, 44, 45, 47, 49–51, 53, 54), and 10 studies included adults (≥ 18 years) (15, 38, 43, 46, 48, 52, 55–58). Regarding asthma severity, 3 studies included patients with mild asthma (52, 54, 57), 8 included patients with mild-to-moderate asthma (39, 43, 47–49, 55, 56, 58), 3 included patients with moderate asthma (38, 40, 51), and 8 did not explicitly report severity (15, 41, 42, 44–46, 53). The included studies were conducted across multiple countries and regions.

#### 3.2.2 Intervention characteristics

The included studies tested a variety of structured physical activity interventions. For subgroup analyses, intervention type was dichotomized into aerobic (running, cycling, swimming, treadmill training, combined aerobic training, etc.) and non-aerobic (yoga, breathing exercises, etc.) according to the principal exercise mechanism. Three studies used multi-arm designs: Carew et al. (39) compared swimming, football, and basketball (yielding 3 comparison units); Shaw et al. (38) compared aerobic exercise, diaphragmatic respiration, and combined exercise (yielding 3 comparison units); and Li et al. (60) compared moderate-intensity continuous training (MCT) with high-intensity interval training (HIIT) (yielding 2 comparison units). Following the Cochrane Handbook recommendation, the control group sample size in these studies was proportionally divided across the intervention arms.

#### 3.2.3 Intervention protocols

Total intervention duration ranged from 6 weeks (39, 47) to 6 months (50); most studies had intervention durations of 8 weeks or less. Weekly frequency ranged from 1 session/week (39, 58) to 7 sessions/week (41, 55, 56). Session duration ranged from 8 min (53) to 80 min (44).

Among the 22 included studies, FEV_1_% predicted was reported by 13 studies, FVC% predicted by 11, PEF% predicted by 7, PEF (L/s) by 5, FEV_1_ (L) by 8, FVC (L) by 6, and PAQLQ by 6.

### 3.3 Risk of bias in included studies

Risk-of-bias assessment results for the 22 included studies are presented in Supplementary Figure 1 (domain-level judgments for each study, traffic light plot) and Supplementary Figure 2 (overall distribution across domains).

In terms of overall risk of bias, 4 studies (18.2%) were rated as low risk (40, 51, 55, 56), 10 studies (45.5%) as having some concerns (15, 41, 42, 44–48, 54, 58), and 8 studies (36.4%) as high risk (38, 39, 43, 49, 50, 52, 53, 57).

The distribution of risk of bias varied substantially across domains. The “measurement of the outcome” domain performed best, with 90.9% of studies rated as low risk, primarily because pulmonary function indices such as FEV_1_, FVC, and PEF were measured using objective spirometry and are unlikely to be affected by assessor subjectivity. In the “missing outcome data” domain, 81.8% of studies were rated as low risk; and in the “deviations from intended interventions” domain, 72.7% were rated as low risk.

However, two domains showed concentrated concerns: (1) in the “randomization process” domain, only 36.4% of studies were rated as low risk and 63.6% had some concerns, mainly because most studies mentioned “random allocation” but did not explicitly describe the random sequence generation method or allocation concealment procedure; (2) in the “selection of the reported result” domain, only 22.7% of studies were rated as low risk, 68.2% had some concerns, and 9.1% were rated as high risk. The concerns in this domain mainly arose from the absence of prospectively registered study protocols in most included studies, making it impossible to verify whether the reported outcomes were consistent with the prespecified analysis plan.

### 3.4 Results of individual studies and syntheses

#### 3.4.1 Primary outcome: FEV_1_% predicted

**FIGURE 2:**
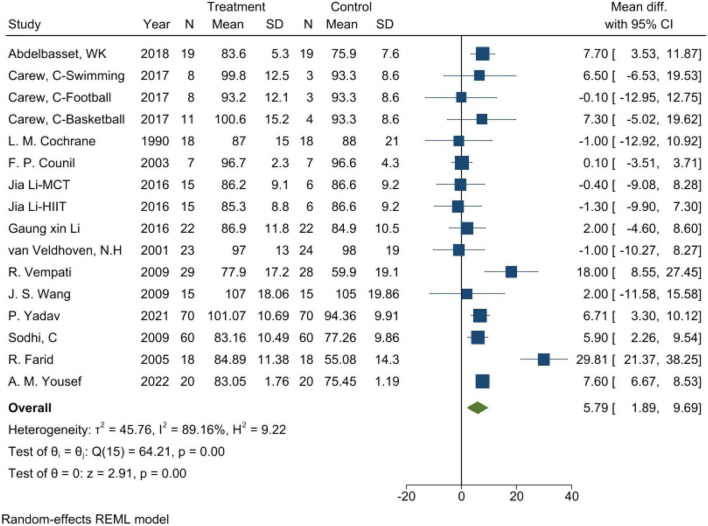
Forest plot of the random-effects meta-analysis of physical activity on FEV_1_% predicted in patients with asthma. Effect estimates are expressed as mean differences with 95% confidence intervals. The pooled estimate was calculated using the restricted maximum likelihood (REML) estimator. Mean diff, mean difference; SD, standard deviation; 95% CI, 95% confidence interval; FEV_1_% predicted, forced expiratory volume in 1 s as a percentage of predicted value; REML, restricted maximum likelihood; MCT, moderate-intensity continuous training; HIIT, high-intensity interval training; AE, aerobic exercise; DR, diaphragmatic respiration; CE, combined exercise.

FEV_1_% predicted was reported by 13 studies contributing 16 effect sizes. The random-effects model (REML estimator) yielded a pooled estimate showing that, compared with the control group, physical activity produced a statistically significant improvement in FEV_1_% predicted in patients with asthma (MD = 5.79, 95% CI: 1.89–9.69, *P* < 0.005), with high heterogeneity (*I*^2^ = 89.16%) (Figure 2). The synthesis was based on 13 studies with the following risk-of-bias distribution: 2 at low risk (40, 51), 8 with some concerns (41, 42, 44, 46–48, 54, 58), and 3 at high risk (39, 43, 53).

#### 3.4.2 Secondary outcome: FVC% predicted

**FIGURE 3:**
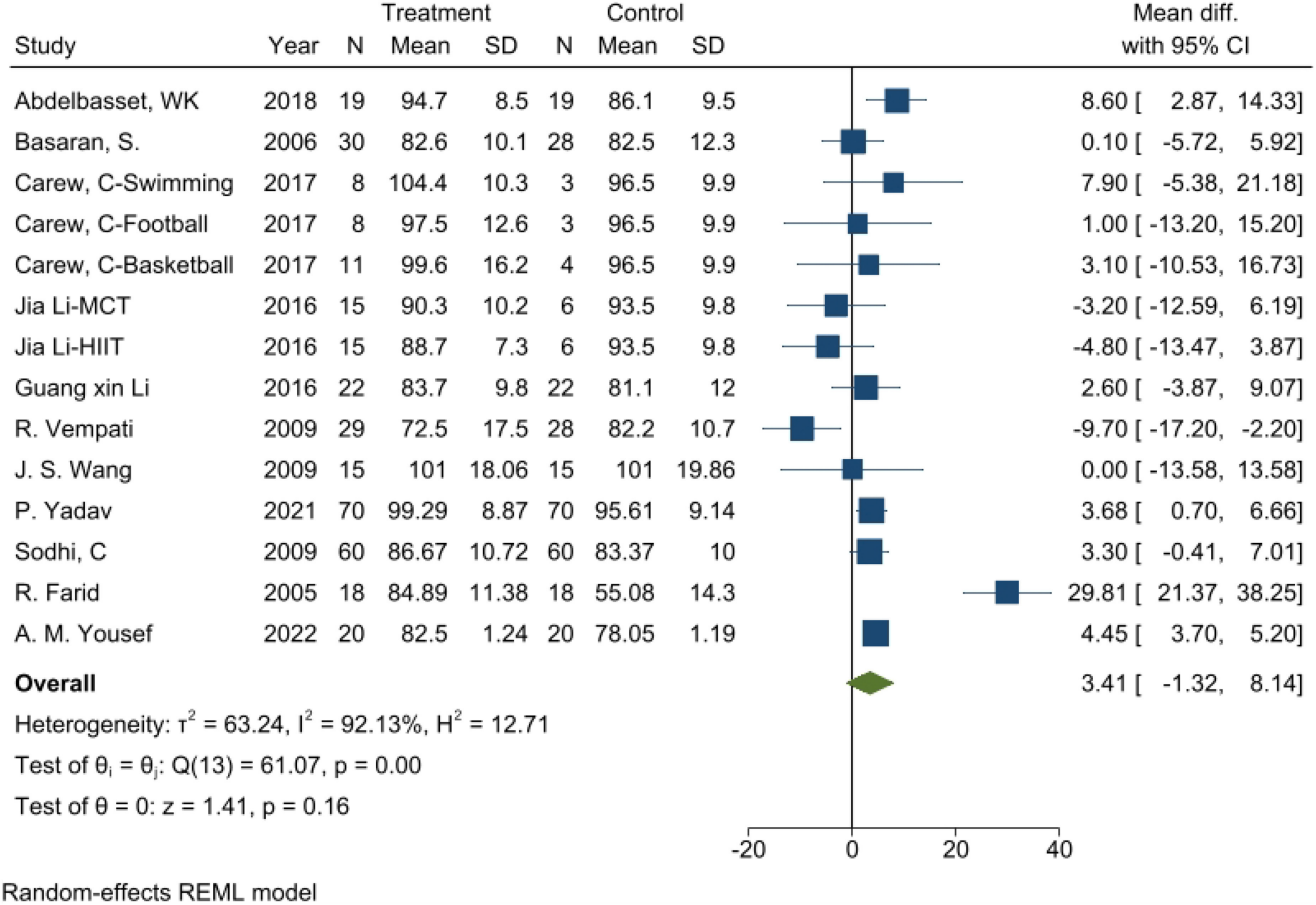
Forest plot of the random-effects meta-analysis of physical activity on FVC% predicted in patients with asthma. Effect estimates are expressed as mean differences with 95% confidence intervals. The pooled estimate was calculated using the restricted maximum likelihood (REML) estimator. Mean diff, mean difference; SD, standard deviation; 95% CI, 95% confidence interval; FVC% predicted, forced vital capacity as a percentage of predicted value; REML, restricted maximum likelihood; MCT, moderate-intensity continuous training; HIIT, high-intensity interval training; AE, aerobic exercise; DR, diaphragmatic respiration; CE, combined exercise.

FVC% predicted was reported by 11 studies contributing 14 effect sizes. The pooled estimate showed that, compared with the control group, physical activity did not produce a statistically significant change in FVC% predicted in patients with asthma (MD = 3.41, 95% CI: −1.32 to 8.14, *P* = 0.16; *I*^2^ = 92.13%) (Figure 3). The synthesis was based on 11 studies with the following risk-of-bias distribution: 2 at low risk (40, 51), 5 with some concerns (41, 42, 46, 54, 58), and 4 at high risk (39, 43, 49, 53).

#### 3.4.3 Secondary outcome: PEF% predicted

PEF% predicted was reported by 7 studies contributing 10 effect sizes. The pooled estimate showed that, compared with the control group, physical activity did not produce a statistically significant change in PEF% predicted in patients with asthma (MD = 5.24, 95% CI: −0.90 to 11.37, *P* = 0.09; *I*^2^ = 90.95%) (forest plot in Supplementary Figure 3). The synthesis was based on 7 studies with the following risk-of-bias distribution: 1 at low risk (40), 3 with some concerns (46, 54, 58), and 3 at high risk (39, 49, 53).

#### 3.4.4 Secondary outcome: PEF (L/s)

PEF was reported in L/s by 5 studies contributing 7 effect sizes. The pooled estimate showed that, compared with the control group, physical activity produced a statistically significant improvement in PEF (L/s) in patients with asthma (MD = 0.47, 95% CI: 0.08–0.85, *P* = 0.02; *I*^2^ = 74.53%) (forest plot in Supplementary Figure 4). The synthesis was based on 5 studies with the following risk-of-bias distribution: 1 at low risk (56), 2 with some concerns (44, 45), and 2 at high risk (38, 52).

#### 3.4.5 Secondary outcome: FEV_1_ (L)

FEV_1_ was reported in liters by 8 studies contributing 10 effect sizes. The pooled estimate showed that, compared with the control group, physical activity did not produce a statistically significant change in FEV_1_ (L) in patients with asthma (MD = 0.06 L, 95% CI: −0.21 to 0.33, *P* = 0.68; *I*^2^ = 93.33%) (forest plot in Supplementary Figure 5). The synthesis was based on 8 studies with the following risk-of-bias distribution: 2 at low risk (55, 56), 3 with some concerns (15, 44, 48), and 3 at high risk (38, 50, 57).

#### 3.4.6 Secondary outcome: FVC (L)

FVC was reported in liters by 6 studies contributing 8 effect sizes. The pooled estimate showed that, compared with the control group, physical activity produced a statistically significant improvement in FVC (L) in patients with asthma (MD = 0.55 L, 95% CI: 0.25–0.84, *P* < 0.001; *I*^2^ = 91.61%) (forest plot in Supplementary Figure 6). The synthesis was based on 6 studies with the following risk-of-bias distribution: 1 at low risk (56), 3 with some concerns (15, 44, 45), and 2 at high risk (38, 57).

#### 3.4.7 Secondary outcome: PAQLQ

The PAQLQ total score was reported by 6 studies contributing 6 effect sizes. The pooled estimate showed that, compared with the control group, physical activity produced a statistically significant improvement in PAQLQ scores in patients with asthma (MD = 1.13, 95% CI: 0.45–1.81, *P* < 0.001; *I*^2^ = 96.14%) (forest plot in Supplementary Figure 7). The synthesis was based on 6 studies with the following risk-of-bias distribution: 2 at low risk (40, 51), 2 with some concerns (41, 45), and 2 at high risk (49, 50).

### 3.5 Meta-regression

To explore potential sources of heterogeneity, we performed univariable random-effects meta-regression analyses for three prespecified continuous covariates—total intervention duration, weekly frequency, and session duration.

For FEV_1_% predicted (*k* = 13 studies, 16 observations), univariable meta-regression showed that none of the three covariates was significantly associated with the effect size. Specifically, total intervention duration (β = −0.030, 95% CI: −1.985 to 1.925, *P* = 0.974), weekly frequency (β = 0.548, 95% CI: −1.832 to 3.074, *P* = 0.647), and session duration (β = 0.043, 95% CI: −0.040 to 0.126, *P* = 0.281) explained very little of the variation in the effect size, with *R*^2^ values of 0.00, 0.00, and 3.59%, respectively. The residual I^2^ values across the univariable models were 86.84, 88.19, and 88.27%, indicating that none of the covariates effectively explained the between-study heterogeneity.

In the bivariable model that simultaneously included intervention duration (β = −0.062, 95% CI: −2.121 to 1.998, *P* = 0.950) and aerobic intervention type (β = 0.885, 95% CI: −8.525 to 10.296, *P* = 0.842), the residual heterogeneity was 87.53%, *R*^2^ = 0.00%, and neither covariate showed a significant moderating effect on the effect size (both *P* > 0.80).

For FVC% predicted (*k* = 11 studies, 14 observations), univariable meta-regression similarly showed no significant moderating effects. Total intervention duration (β = 0.341, 95% CI: −3.089 to 3.772, *P* = 0.831), weekly frequency (β = −0.572, 95% CI: −4.326 to 3.181, *P* = 0.744), and session duration (β = −0.017, 95% CI: −0.129 to 0.094, *P* = 0.74) explained essentially none of the effect-size variability (*R*^2^ = 0.00% for all models), with residual I^2^ values of 87.97, 92.69, and 92.61%, respectively.

In the bivariable model, intervention duration (β = 0.041, 95% CI: −3.534 to 3.617, *P* = 0.98) and aerobic intervention type (β = −5.371, 95% CI: −17.841 to 7.097, *P* = 0.36) jointly yielded a residual heterogeneity of 87.23% and *R*^2^ = 0.00%, with neither covariate showing a significant moderating effect. Detailed results are presented in Supplementary Tables 3, 4.

### 3.6 Subgroup analyses

Prespecified subgroup analyses were conducted for FEV_1_% predicted and FVC% predicted, with results summarized in Supplementary Table 5. No between-subgroup comparison reached statistical significance (all *P* > 0.05), indicating that the available evidence is insufficient to confirm that participant age, total intervention duration, weekly frequency, session duration, or exercise type act as effect moderators when treated as categorical variables.

In the age subgroup, for FEV_1_% predicted, the children/adolescent subgroup ( < 18 years, *k* = 13) showed a pooled effect of MD = 3.81% (95% CI: 1.30–6.31, *P* < 0.001; *I*^2^ = 60.07%), and the adult subgroup ( ≥ 18 years, *k* = 3) showed MD = 17.48% (95% CI: 3.59–31.37, *P* = 0.013; *I*^2^ = 91.46%); the between-subgroup difference did not reach statistical significance (Qb = 3.60, *P* = 0.06). For FVC% predicted, the children/adolescent subgroup (k = 11) showed MD = 3.58% (95% CI: 1.93–5.22, *P* < 0.001; *I*^2^ = 18.28%), and the adult subgroup (*k* = 3) showed MD = 7.69% (95% CI: −14.84 to 30.22, *P* = 0.50; *I*^2^ = 97.31%); the between-subgroup difference was not significant (Qb = 0.13, *P* = 0.72).

In the intervention type subgroup, for FEV_1_% predicted, the non-aerobic subgroup (*k* = 8) showed a pooled effect of MD = 7.40% (95% CI: 6.54–8.26, *P* < 0.001; *I*^2^ = 0.00%), and the aerobic subgroup (k = 7) showed MD = 5.31% (95% CI: −2.82 to 13.44, *P* = 0.20; *I*^2^ = 91.23%); the between-subgroup difference was not significant (Qb = 0.25, *P* = 0.62). For FVC% predicted, the aerobic subgroup (*k* = 6) showed MD = 5.52% (95% CI: −4.49 to 15.52, *P* = 0.28; *I*^2^ = 91.52%), and the non-aerobic subgroup (*k* = 8) showed MD = 2.11% (95% CI: −1.36 to 5.58, *P* = 0.23; *I*^2^ = 73.24%); the between-subgroup difference was not significant (Qb = 0.40, *P* = 0.53).

In the intervention duration subgroup, for FEV_1_% predicted, the long-duration subgroup ( > 8 weeks, *k* = 5) showed a pooled effect of MD = 7.42% (95% CI: 6.55– 8.30, *P* < 0.001; *I*^2^ = 0.00%), and the short-duration subgroup ( ≤ 8 weeks, k = 11) showed MD = 6.35% (95% CI: 0.53–12.18, *P* = 0.033; *I*^2^ = 85.10%); the between-subgroup difference was not significant (Qb = 0.13, *P* = 0.72). For FVC% predicted, the long-duration subgroup (*k* = 3) showed MD = 4.47% (95% CI: 3.75–5.20, *P* < 0.001; *I*^2^ = 0.00%), and the short-duration subgroup (*k* = 11) showed MD = 2.69% (95% CI: −3.60 to 8.98, *P* = 0.40; *I*^2^ = 85.06%).

In the weekly frequency subgroup, for FEV_1_% predicted, the high-frequency subgroup ( > 3 sessions/week, *k* = 3) showed a pooled effect of MD = 7.85% (95% CI: −1.72 to 17.41, *P* = 0.10; *I*^2^ = 81.77%), and the low-frequency subgroup ( ≤ 3 sessions/week, *k* = 13) showed MD = 5.28% (95% CI: 0.81–9.76, *P* = 0.020; *I*^2^ = 89.30%); the between-subgroup difference was not significant (Qb = 0.23, *P* = 0.63). For FVC% predicted, the high-frequency subgroup (k = 3) showed MD = −2.46% (95% CI: −10.67 to 5.76, *P* = 0.56; *I*^2^ = 79.39%), and the low-frequency subgroup (*k* = 10) showed MD = 5.23% (95% CI: −0.15 to 10.61, *P* = 0.06; *I*^2^ = 90.29%); the between-subgroup difference was not significant (Qb = 2.35, *P* = 0.12).

In the session duration subgroup, for FEV_1_% predicted, the long-session subgroup ( ≥ 45 min, *k* = 6) showed a pooled effect of MD = 5.12% (95% CI: 0.42–9.81, *P* = 0.033; *I*^2^ = 77.05%), and the short-session subgroup ( < 45 min, *k* = 10) showed MD = 6.09% (95% CI: 0.28–11.90, *P* = 0.040; *I*^2^ = 88.63%); the between-subgroup difference was not significant (Qb = 0.07, *P* = 0.80). For FVC% predicted, the long-session subgroup (*k* = 4) showed MD = −0.06% (95% CI: −6.39 to 6.28, *P* = 0.99; *I*^2^ = 82.31%), and the short-session subgroup (*k* = 10) showed MD = 5.04% (95% CI: −1.09 to 11.17, *P* = 0.11; I^2^ = 89.74%); the between-subgroup difference was not significant (Qb = 1.28, *P* = 0.26).

### 3.7 Reporting bias assessment

Publication bias was formally assessed for the two outcomes with ≥ 10 included studies (FEV_1_% predicted and FVC% predicted).

For FEV_1_% predicted, visual inspection of the funnel plot revealed no apparent asymmetry. Egger’s regression test did not detect a significant small-study effect (β_1_ = −0.27, *P* = 0.800). The trim-and-fill analysis imputed 5 potentially missing studies on the right side of the funnel plot; the adjusted pooled effect was MD = 8.288 (95% CI: 4.624–11.951), higher than the observed estimate (MD = 5.791, 95% CI: 1.891–9.691). The direction of the effect and the overall conclusion were unchanged after imputation.

For FVC% predicted, visual inspection of the funnel plot likewise revealed no apparent asymmetry. Egger’s regression test did not detect a significant small-study effect (β_1_ = −0.16, *P* = 0.899). The trim-and-fill analysis imputed 5 potentially missing studies on the right side of the funnel plot; the adjusted pooled effect was MD = 7.160 (95% CI: 2.504–11.816), higher than the observed estimate (MD = 3.409, 95% CI: −1.320–8.137). Notably, the adjusted 95% CI for FVC% predicted no longer crossed the null, suggesting that if larger-effect studies have been selectively unpublished, the true effect may be statistically significant.

Taken together, the results of Egger’s test and the trim-and-fill analysis indicated no statistically significant evidence of publication bias for either outcome (funnel plots in Supplementary Figures 8, 9).

### 3.8 Sensitivity analyses

#### 3.8.1 FEV_1_% predicted

Leave-one-out analysis showed that the pooled effect ranged from MD = 4.67–6.26 after omitting each effect size in turn, and all leave-one-out estimates remained statistically significant (all *P* ≤ 0.010), indicating the robustness of the primary analysis. Omission of Farid 2005 yielded the largest decrease in the pooled estimate (MD = 4.67, 95% CI: 2.39–6.95), but did not change the direction of the conclusion (Supplementary Figure 10).

Comparison of heterogeneity variance estimators: the DL estimator yielded MD = 5.85 (95% CI: 3.03–8.67; *I*^2^ = 76.64%) (Supplementary Figure 12); the PM estimator yielded MD = 5.79 (95% CI: 1.84–9.74; *I*^2^ = 89.49%) (Supplementary Figure 13). The three estimators produced consistent results in terms of the direction and statistical significance of the pooled effect.

Based on the leave-one-out analysis, we additionally re-pooled the data after simultaneously excluding the two most influential studies, Farid 2005 and Yousef 2022 (12 studies, 14 effect sizes), which yielded MD = 4.10 (95% CI: 1.53–6.67, *P* < 0.001; *I*^2^ = 48.73%). The heterogeneity decreased substantially to a moderate level, while the direction and statistical significance of the pooled effect were preserved.

#### 3.8.2 Other secondary outcomes

For FVC% predicted, leave-one-out analysis showed that the pooled effect ranged from MD = 2.22–4.49 after omitting each effect size in turn, and none of the leave-one-out estimates reached statistical significance (all *P* > 0.05), consistent with the primary analysis. Omission of Farid 2005 yielded the largest decrease in the pooled estimate (MD = 2.22, 95% CI: 0.004–4.43).

Comparison of heterogeneity variance estimators for FVC% predicted: the DL estimator yielded MD = 3.47 (95% CI: 0.38–6.57; *I*^2^ = 78.71%, *P* = 0.032) (Supplementary Figure 14); the PM estimator yielded MD = 3.41 (95% CI: −1.31 to 8.13; *I*^2^ = 92.09%, *P* = 0.162) (Supplementary Figure 15). Notably, the DL estimator yielded a statistically significant pooled effect for FVC% predicted (*P* = 0.032), whereas neither the REML nor the PM estimator did, indicating that statistical inference for this outcome is sensitive to the choice of estimator and should be interpreted with caution.

Leave-one-out analyses were also performed for PEF% predicted, PEF (L/s), FEV_1_ (L), FVC (L), and PAQLQ (Supplementary Figure 11):

PEF% predicted (*k* = 7, 10 effect sizes): the pooled effect ranged from MD = 3.95–5.37 after omitting each effect size; all leave-one-out estimates were consistent with the primary analysis.

PEF (L/s) (*k* = 5, 7 effect sizes): the pooled effect ranged from MD = 0.44–0.68; all leave-one-out estimates were consistent with the primary analysis.

FEV_1_ (L) (*k* = 8, 10 effect sizes): the pooled effect ranged from MD = −0.01 to 0.11; all leave-one-out estimates were consistent with the primary analysis.

FVC (L) (*k* = 6, 8 effect sizes): the pooled effect ranged from MD = 0.45–0.62; all leave-one-out estimates were consistent with the primary analysis.

PAQLQ (*k* = 6, 6 effect sizes): the pooled effect ranged from MD = 0.79–1.31; all leave-one-out estimates were consistent with the primary analysis.

Across all secondary outcomes, leave-one-out analyses indicated that the conclusions of the primary analyses were robust.

### 3.9 Certainty of evidence

The certainty of evidence for four outcomes was assessed using the GRADE framework; detailed results are presented in Supplementary Table 6.

The certainty of evidence for FEV_1_% predicted was rated as low. The starting level was high (RCTs), and downgrades were applied for risk of bias (−1 level; only 15.4% of studies were at low overall risk of bias) and inconsistency (−1 level; *I*^2^ = 89.16%, although subgroup analyses partially identified heterogeneity sources). No downgrade was applied for indirectness, imprecision, or publication bias.

The certainty of evidence for FVC% predicted was rated as very low. Downgrades were applied for risk of bias (−1 level), inconsistency (−2 levels; *I*^2^ = 92.13%, with subgroup analyses unable to explain heterogeneity), and imprecision (−1 level; 95% CI crosses the null), yielding a total downgrade of 4 levels.

The certainty of evidence for PEF% predicted was rated as very low. Downgrades were applied for risk of bias (−1 level; only 14.3% of studies at low risk), inconsistency (−2 levels; *I*^2^ = 90.95%), and imprecision (−1 level; 95% CI crosses the null, *P* = 0.09), yielding a total downgrade of 4 levels. With fewer than 10 studies, a formal publication bias test was not feasible.

The certainty of evidence for PAQLQ was rated as very low. Downgrades were applied for risk of bias (−1 level) and inconsistency (−2 levels; *I*^2^ = 96.14%), yielding a total downgrade of 3 levels. Although the pooled effect was statistically significant with a 95% CI that did not cross the null, the high heterogeneity and indirectness limited the strength of the evidence.

Overall, the current evidence statistically supports a positive effect of physical activity on FEV_1_% predicted and PAQLQ in patients with asthma, but the overall certainty of evidence is low, influenced by risk of bias and heterogeneity. More rigorously designed and standardly reported RCTs are needed in the future to upgrade the certainty of evidence.

## 4 Discussion

### 4.1 Summary of main findings and comparison with previous evidence

This systematic review and meta-analysis evaluated the effects of physical activity on pulmonary function and quality of life in patients with asthma, drawing on RCTs that spanned a broad age range. The core finding presents a systematic, outcome-specific pattern: physical activity yielded statistically significant effects on FEV_1_% predicted and PAQLQ scores, but did not reach statistical significance for FVC% predicted or PEF% predicted. More notably, the three pulmonary function indices showed a strikingly consistent cross-scale discordance—FEV_1_ was significant only on the % predicted scale, whereas FVC and PEF were significant only when expressed in absolute units (liters, liters per second). This cross-outcome, cross-scale systematic discordance is a methodologically important finding that has not been systematically discussed in the existing literature.

The incremental contribution of the present review must be situated within the boundaries of prior syntheses. The meta-analysis by Hansen et al. was confined to aerobic exercise in adults and reported small but favorable effects on pulmonary function (21); the reviews by Wu et al. and Lu et al. were confined to pediatric populations, with the former finding that physical training significantly improved FVC% predicted but not FEV_1_% predicted (26), and the latter network meta-analysis observing an effect on FEV_1_% predicted only within a combined endurance-and-breathing subgroup (27); a 2025 umbrella review synthesized conclusions across multiple meta-analyses (60), but the constituent meta-analyses varied in their operationalization of pulmonary function outcomes and none systematically distinguished percentage-predicted from absolute-value expressions. The incremental contributions of the present review relative to these prior syntheses are threefold. First, by pooling the same physiological domain on two expression scales within a single synthetic framework, and by prespecifying the percentage-predicted scale as the primary outcome, we identified a cross-scale discordance—the opposing significance patterns of FEV_1_ and FVC across the two scales—that itself constitutes an independent contribution to the methodological discourse in this field. Second, by prespecifying subgroup analyses to systematically test the moderating effects of intervention characteristics on effect consistency, we identified non-aerobic exercise type and longer intervention duration as potential moderators of effect robustness, generating hypotheses for clinical prescription. Third, by combining leave-one-out sensitivity analyses with multiple heterogeneity variance estimators, we traced the overall high heterogeneity to specific studies and intervention characteristics, providing actionable directions for subsequent research. A recent meta-analysis based on adherence to ACSM recommendations similarly found that interventions conforming to standardized exercise prescriptions yielded greater improvements in FVC and quality of life (60), consistent in direction with our subgroup signal regarding the influence of intervention structuredness on effect consistency.

The effect of physical activity on quality of life in patients with asthma has shown comparatively good consistency in the prior literature. Pulmonary rehabilitation studies in adults have reported significant improvements in the Asthma Quality of Life Questionnaire (AQLQ) (61); aerobic-based pediatric pulmonary rehabilitation programs have likewise demonstrated significant improvements in quality of life (62); an overview that integrated multiple systematic reviews indicated that physical activity interventions consistently improve asthma-related quality of life across different exercise modalities (63); and physical activity interventions in patients with severe asthma have improved health-related quality of life (64).

In the present review, the quality-of-life pooled estimate was derived from the PAQLQ, and all original studies contributing to this synthesis enrolled children or adolescents, matching exactly the population for which the PAQLQ has been validated. The magnitude of PAQLQ improvement observed in our review lies at the higher end of the existing pediatric asthma literature, suggesting that physical activity confers a substantive benefit on health-related quality of life in this population; however, given the substantial between-study heterogeneity and the potential for expectancy effects on self-reported outcomes in unblinded exercise interventions, this finding should be interpreted with caution.

Several reviews of specific intervention modalities help contextualize the heterogeneity observed in our pooled estimates. In adults, a network meta-analysis reported that relaxation training, combined breathing-and-aerobic training, and yoga produced the largest effects on FEV_1_, FVC, and PEF, respectively (28); meta-analyses specifically focused on yoga (23, 24), breathing training (65), and inspiratory muscle training (66) have all reported directionally positive but heterogeneous effects on pulmonary function. In contrast, reviews focused on single intervention types such as swimming (22), water-based exercise (67), and high-intensity interval training (25) have observed relatively limited or non-significant effects on pulmonary function. A recent head-to-head RCT comparing constant-load exercise with high-intensity interval training in moderate-to-severe asthma demonstrated comparable improvements in aerobic fitness between the two modalities (68), suggesting that the principal driver of pulmonary functional benefit may be the structured, sustained nature of the training rather than its specific intensity profile.

### 4.2 Outcome specificity and cross-scale discordance: physiological interpretation and sources of heterogeneity

The most salient finding of our review is the discordant pattern of statistical significance across different expressions of the same physiological domain. This phenomenon warrants interpretation at both the physiological and methodological levels.

From a physiological perspective, asthma is fundamentally an obstructive disease characterized by chronic airway inflammation, airway hyperresponsiveness, and variable expiratory airflow limitation (69). FEV_1_ is widely regarded as the single most sensitive spirometric index of airway obstruction (69). Although FVC is conventionally a volume index, in the context of asthma it is not a purely volumetric measure—patients with severe asthma can develop dynamic hyperinflation during exercise, indicating that airway obstruction also influences volume-related measurements through gas-trapping mechanisms (70); the distinction between FVC and FEV_1_ in asthma is therefore less clear-cut than the classical obstructive–restrictive framework would suggest. Within this framework, one plausible interpretation is that the effects of physical activity on pulmonary function in patients with asthma are mediated primarily through airway-level mechanisms—including reductions in bronchial hyperresponsiveness and airway inflammation—mechanisms whose direct action on airflow indices (FEV_1_) is relatively well established (71), while their effect on volume-related indices (FVC) is conceptually less direct. The pattern we observed—significant improvement in FEV_1_% predicted but not in FVC% predicted—is directionally consistent with this inference; however, because the included studies generally did not report airway inflammation markers, bronchial responsiveness, or small-airway function indices, this should be regarded as a physiological interpretation rather than a definitive explanation, and remains to be tested in dedicated trials incorporating mechanistic assessments.

When interpreting the cross-scale discordance, it is necessary to return to our prespecified outcome framework: percentage-predicted indices were designated as primary outcomes, a choice aligned with the standardization framework of spirometry reference equations (72), aimed at removing the confounding influence of anthropometric variables—height, age, sex, and ethnicity—on absolute values. On the primary-outcome scale, all three pulmonary function indices showed a consistent pattern: FEV_1_% predicted improved significantly, while FVC% predicted and PEF% predicted did not. This pattern aligns with the core pathophysiological expectation in asthma—airflow limitation is the hallmark functional change, and FEV_1_ is its most sensitive spirometric index (69); even though FVC is influenced by gas trapping in asthma (70), its response to exercise interventions is conceptually less direct than that of FEV_1_. The significant findings observed for FVC in liters and PEF in L/s should be interpreted as secondary supportive information from the absolute-value scale—because absolute values are confounded by anthropometric variables and our sample spans a wide age range from children to middle-aged adults, the significant findings on these scales may partly reflect sample characteristics rather than the intervention effect *per se*. Recent methodological work also supports the rationale for using relative-scale metrics as the primary analysis in continuous spirometric outcome syntheses (73).

The FEV_1_-dominant pattern of response to physical activity finds directional support in the broader exercise-mechanism literature. Exercise training has been shown to reduce bronchial hyperresponsiveness and exercise-induced bronchoconstriction—both airway-level phenomena—with these improvements appearing in tandem with improvements in symptoms and quality of life (71). Inspiratory muscle training can improve maximal inspiratory pressure—a marker of respiratory muscle strength—but does not consistently produce changes in absolute FEV_1_ or FVC (66), suggesting that exercise effects in asthma may be mediated mainly through airway-level mechanisms. Yoga and breathing-based interventions have likewise been shown to improve asthma-related quality of life and pulmonary function indices, with proposed mechanisms including modulation of breathing patterns, reduction of airway hyperresponsiveness, and improvement of respiratory muscle function (65, 74).

These mechanistic considerations resonate with the direction of our subgroup analyses. For FEV_1_% predicted, the non-aerobic subgroup (*k* = 8, including yoga, breathing exercises, and ball sports) showed a pooled effect of MD = 7.40% with within-subgroup *I*^2^ = 0.00%, while the aerobic subgroup (*k* = 7) showed MD = 5.31% with *I*^2^ = 91.23%; the subgroup with intervention duration > 8 weeks (*k* = 5) showed MD = 7.42% with *I*^2^ = 0.00%, while the short-duration subgroup (*k* = 11) showed *I*^2^ = 85.10%. For FVC% predicted, the long-duration subgroup (*k* = 3) likewise showed *I*^2^ = 0.00%, but with a smaller number of studies. It must be emphasized that none of the between-subgroup Qb comparisons reached statistical significance (*P*-values ranged from 0.62 to 0.80 for FEV_1_% predicted subgroups, and from 0.12 to 0.72 for FVC% predicted), and the non-aerobic and long-duration subgroups had limited numbers of studies (*k* = 3–8). Under these conditions, the low within-subgroup I^2^ estimates may largely reflect estimation instability arising from limited study counts rather than a genuine reduction in heterogeneity.

The meta-regression analyses provide an independent inferential perspective on these subgroup observations. Univariable meta-regression showed that none of total intervention duration, weekly frequency, session duration, or intervention type (aerobic vs. non-aerobic) was significantly associated with the effect size for either FEV_1_% predicted or FVC% predicted (all *P* > 0.20), with *R*^2^ values generally at 0.00%; bivariable models simultaneously including intervention duration and intervention type yielded the same conclusion (all *P* > 0.40). Meta-regression and subgroup analysis assess different statistical properties—the former evaluates the continuous association between covariates and effect size, while the latter compares effect estimates and stability across strata—but neither approach provided consistent evidence that intervention characteristics modify the effect size in our data. Accordingly, the subgroup observation that “non-aerobic exercise plus longer duration corresponds to lower within-subgroup heterogeneity” must be classified strictly as hypothesis-generating: it suggests priority directions for future research—namely, direct head-to-head trials comparing structured breathing-based interventions with standardized aerobic protocols, paired with assessments of airway physiology—rather than supporting any interpretation that extends beyond what the statistical tests can sustain. Whether this subgroup signal reflects a real moderating effect (involving putative mechanisms such as respiratory muscle training, breathing-pattern modulation, or sustained anti-inflammatory adaptation) or is merely a statistical artifact of small subgroups composed of few studies cannot be adjudicated from our data alone.

Sensitivity analyses identified Farid 2005 and Yousef 2022 as the two studies most influential on the FEV_1_% predicted pooled estimate; simultaneous removal of these two studies substantially reduced heterogeneity from *I*^2^ = 89.16% to *I*^2^ = 48.73% (moderate), while preserving the direction and statistical significance of the pooled effect (MD = 4.10, 95% CI: 1.53–6.67). This result does not constitute grounds for excluding these studies from the primary analysis; rather, it indicates that the pooled conclusion is robust to between-study variation and that heterogeneity is concentrated in a small number of trials—a configuration that strengthens rather than weakens inferences drawn from the pooled estimate. In addition, the three heterogeneity variance estimators (REML, DL, PM) yielded consistent conclusions in terms of direction and significance for FEV_1_% predicted, but for FVC% predicted, the DL estimator produced a statistically significant result (*P* = 0.032) while neither REML nor PM did, indicating that statistical inference for FVC% predicted is sensitive to the choice of estimator and should be interpreted with caution.

The meta-regression analyses provide an independent inferential perspective on these subgroup observations. Univariable meta-regression showed that none of total intervention duration, weekly frequency, session duration, or intervention type (aerobic vs. non-aerobic) was significantly associated with the effect size for either FEV_1_% predicted or FVC% predicted (all *P* > 0.20), with *R*^2^ values generally at 0.00%; bivariable models simultaneously including intervention duration and intervention type yielded the same conclusion (all *P* > 0.40). Meta-regression and subgroup analysis assess different statistical properties—the former evaluates the continuous association between covariates and effect size, while the latter compares effect estimates and stability across strata—but neither approach provided consistent evidence that intervention characteristics modify the effect size in our data. Accordingly, the subgroup observation that “non-aerobic exercise plus longer duration corresponds to lower within-subgroup heterogeneity” must be classified strictly as hypothesis-generating: it suggests priority directions for future research—namely, direct head-to-head trials comparing structured breathing-based interventions with standardized aerobic protocols, paired with assessments of airway physiology—rather than supporting any interpretation that extends beyond what the statistical tests can sustain. Whether this subgroup signal reflects a real moderating effect (involving putative mechanisms such as respiratory muscle training, breathing-pattern modulation, or sustained anti-inflammatory adaptation) or is merely a statistical artifact of small subgroups composed of few studies cannot be adjudicated from our data alone.

Sensitivity analyses identified Farid 2005 and Yousef 2022 as the two studies most influential on the FEV_1_% predicted pooled estimate; simultaneous removal of these two studies substantially reduced heterogeneity from *I*^2^ = 89.16% to *I*^2^ = 48.73% (moderate), while preserving the direction and statistical significance of the pooled effect (MD = 4.10, 95% CI: 1.53–6.67). This result does not constitute grounds for excluding these studies from the primary analysis; rather, it indicates that the pooled conclusion is robust to between-study variation and that heterogeneity is concentrated in a small number of trials—a configuration that strengthens rather than weakens inferences drawn from the pooled estimate. In addition, the three heterogeneity variance estimators (REML, DL, PM) yielded consistent conclusions in terms of direction and significance for FEV_1_% predicted, but for FVC% predicted, the DL estimator produced a statistically significant result (*P* = 0.032) while neither REML nor PM did, indicating that statistical inference for FVC% predicted is sensitive to the choice of estimator and should be interpreted with caution.

### 4.3 Limitations

This review has limitations at multiple levels, including limitations of the included evidence itself and limitations of the review process.

#### 4.3.1 Limitations of the included evidence

The methodological quality of the included trials was moderate overall. Most studies were rated as having “some concerns” or “high risk of bias” on the Cochrane RoB 2 tool, with concerns most concentrated in the randomization process and selection of the reported result domains. Most trials did not provide sufficient detail on allocation concealment, and the majority were conducted before trial registration practices became widespread, so the possibility of selective outcome reporting cannot be ruled out. In addition, the nature of exercise interventions makes participant blinding inherently infeasible, and most trials did not blind outcome assessors either—this is a structural limitation of exercise-intervention RCTs rather than a deficiency in study conduct, but for patient-reported outcomes (such as the PAQLQ), the influence of expectancy effects cannot be fully excluded. The impact on objective spirometric indices is comparatively smaller, and the directionally consistent improvements in objective (FEV_1_% predicted) and patient-reported (PAQLQ) outcomes observed in our review provide some reassurance that the effects are not entirely driven by expectancy bias. These methodological reporting patterns—rather than necessarily the actual conduct of the trials—are the principal basis for the GRADE downgrades. The number of studies judged to be at low overall risk of bias was insufficient to support a sensitivity analysis restricted to low-bias studies. Consequently, the findings of this review should be interpreted in the context of low-to-very-low certainty of evidence.

The clinical heterogeneity across included trials was substantial. Participants spanned a wide age range; asthma severity was inconsistently reported and largely confined to mild-to-moderate disease; and interventions varied in type, duration, frequency, and degree of supervision. The PAQLQ synthesis was based on six studies, all in pediatric or adolescent populations—matching exactly the population for which the PAQLQ has been validated—so this outcome is free from scale–population mismatch as a source of indirectness; however, this also means that our direct quantitative evidence regarding quality of life cannot be extrapolated to adults with asthma. The prespecified subgroup analyses for asthma severity and risk of bias could not be performed because the number of trials reporting these variables was insufficient, limiting our ability to characterize “who benefits most from which intervention under which methodological conditions.”

The pulmonary function outcomes were reported inconsistently across studies—the same physiological domain was expressed as absolute values in some studies and as percentage-predicted in others—which is a fundamental problem that constrained the way outcomes could be pooled. Although we addressed this by pooling each scale separately and prespecifying percentage-predicted indices as the primary outcome, the underlying reporting inconsistency still limited the precision and comparability of the pooled estimates, reflecting a field-wide absence of standardized reporting that cannot be fully remedied at the synthesis stage.

Regarding publication bias, we performed formal statistical assessment for the two outcomes with ≥ 10 studies (FEV_1_% predicted and FVC% predicted); outcomes with fewer than 10 studies could not be assessed in this way. Visual inspection of funnel plots revealed no apparent asymmetry, and Egger’s test was not statistically significant for either outcome. The trim-and-fill analysis imputed potentially missing studies on the right side of the funnel plot for both outcomes, with the imputed pooled estimates exceeding the observed estimates; this direction implies that if selective publication has occurred, the missing studies are those with larger rather than smaller effects, and the current pooled estimates are more likely to underestimate than overestimate the true effect. Nevertheless, given the systematic background of positive results being more likely to be published in exercise-intervention research (75), and the small sample sizes of most included trials, the influence of publication bias on the pooled estimates cannot be entirely excluded.

#### 4.3.2 Limitations of the review process

Several limitations at the review level merit mention. First, we did not re-search CNKI in the updated search, on the rationale that the proportion of Chinese-language studies meeting our inclusion criteria during the initial search was low; this decision may have resulted in incomplete coverage of recent Chinese-language literature. Second, although our inclusion criteria required a minimum intervention duration of 6 weeks, the duration distribution among included studies was right-skewed, with most trials lasting 8 weeks or less; evidence regarding longer-term physical activity programs—for which biological adaptations might be more fully developed—therefore remains limited. Third, three included trials used multi-arm designs in which multiple intervention arms shared a single control group; we addressed the resulting unit-of-analysis issue by proportionally dividing the control group sample size across arms following the Cochrane Handbook recommendation, but although this is standard practice, it may slightly underestimate the statistical precision of the control-group estimate. Fourth, the covariates included in our meta-regression were limited to intervention-level characteristics (total duration, weekly frequency, session duration, and exercise type), because participant-level variables (such as mean age and baseline FEV_1_) could not be constructed uniformly across all studies—not all studies separately reported these values for the intervention and control groups—so participant-level effect modification is incompletely characterized in this review.

### 4.4 Implications for clinical practice and future research

The findings of this review align with current guideline recommendations, further reinforcing the role of physical activity as an adjunctive intervention in asthma management. The Global Initiative for Asthma (GINA) recommends that patients with asthma engage in regular physical activity for overall health benefits and acknowledges a small but favorable effect on asthma symptom control and pulmonary function, while noting that there is insufficient evidence to prioritize any particular form of physical activity (69). A recent European Academy of Allergy and Clinical Immunology (EAACI) position statement emphasized that, in the design of individualized asthma management plans, exercise should be considered alongside pharmacotherapy, and that clinically actionable, asthma-specific exercise prescriptions remain underdeveloped in routine care (17). Pediatric asthma–specific guidance documents likewise emphasize individualized exercise programs that account for asthma control status, fitness level, exercise habits, and environmental factors, positioning exercise as an indispensable—rather than optional—component of asthma management (76, 77). Building on these recommendations, our review provides quantitative evidence: the effect of physical activity is most consistently demonstrable on FEV_1_% predicted and PAQLQ scores; at the subgroup level, programs employing non-aerobic exercise or longer intervention durations exhibit greater robustness in improving FEV_1_% predicted, but this signal is hypothesis-generating and does not yet constitute grounds for recommending specific intervention types.

Several practical implications follow. First, when counseling patients with asthma about exercise, clinicians can convey that the evidence for benefits on airflow indices and quality of life is relatively consistent, whereas the effect on volume-related indices is currently unclear; patient-reported quality-of-life benefits are primarily supported by evidence in children and adolescents, and benefits in adult patients should be referenced to an independent evidence body using adult-validated instruments such as the AQLQ. Second, given that physical inactivity remains widespread among patients with asthma and is itself a modifiable risk factor for adverse outcomes (78), the central clinical question is not whether to recommend physical activity, but how to design and implement it in a sustainable way—consistent with current calls for individualized prescription and structured implementation (17). Third, given the overall low certainty of the included evidence and the short intervention duration of most trials, clinical recommendations should be based on individual patient preferences, accessibility, and adherence, avoiding overly specific prescription decisions based on the current subgroup analyses.

Future research should prioritize several directions. First, pulmonary function outcomes should be reported on the percentage-predicted scale with the reference equation explicitly specified, with absolute values reported as supplementary rather than substitutive—our observation that FEV_1_, FVC, and PEF show opposing significance patterns on the two expression scales highlights the urgency of standardizing reporting. Second, clinical trials should be prospectively registered and report all primary and secondary outcomes, as well as full details of randomization, allocation concealment, and outcome data—these are the principal contributors to GRADE downgrades in our review. Third, the pattern we observed (“non-aerobic exercise plus longer duration corresponds to lower within-subgroup heterogeneity”) should be tested in direct head-to-head trials—comparing structured breathing-based interventions with aerobic training with adequate sample size and intervention duration; ideally, such validation trials should incorporate airway inflammation markers, bronchial responsiveness, or small-airway function indices to characterize whether the observed effects reflect specific biological pathways rather than relying solely on clinical endpoints. Fourth, given the structural infeasibility of blinding in exercise interventions, future trials should adopt attention-matched controls where possible to separate expectancy effects from genuine intervention effects, particularly when assessing patient-reported outcomes. Fifth, multi-arm trials with shared control groups should plan adequate sample size at the design stage to support unbiased pairwise comparisons. Finally, future trials should report participant-level age and baseline pulmonary function in a form that supports study-level meta-regression—that is, group-specific means and standard deviations—so that subsequent syntheses can more reliably characterize individual effect modification.

## 5 Conclusion

This systematic review and meta-analysis of 22 RCTs in 1,280 patients with asthma indicates that physical activity is associated with improved FEV_1_% predicted and PAQLQ scores, with directionally consistent benefits observed for absolute-unit pulmonary function indices. The systematic cross-scale discordance across FEV_1_, FVC, and PEF underscores the importance of standardized outcome reporting in future trials. The overall certainty of evidence is low, primarily due to risk of bias and substantial heterogeneity, indicating that more rigorously designed and standardly reported RCTs are needed to upgrade the certainty of evidence and to clarify the optimal modality and dose of physical activity in asthma management.

## Data Availability

The original contributions presented in this study are included in the article and Supplementary material. Further inquiries can be directed to the corresponding author.
