# Supplementary material for: Effect of physical activity on pulmonary function and quality of life in asthma patients: a systematic review and meta-analysis
Source: Front Med (Lausanne). 2026 Jun 12;13:1848571. doi: 10.3389/fmed.2026.1848571 (PMC13303988; doi:10.3389/fmed.2026.1848571)
Supplement: Supplementary file 1 [file Supplementary_file_1.docx]

# Supplementary Table 1. Literature search strategies in the six electronic databases.

| **Database 1: Ovid MEDLINE(R) ALL** | | |
| --- | --- | --- |
| **No.** | **Search Strategy** | **Results** |
| 1 | Exercise Therapy/ or Exercise/ | 212,292 |
| 2 | (exercise or exercises or exercise, physical or exercises, physical or physical exercises or exercise, aerobic or aerobic exercises or exercises, aerobic or exercise, isometric or exercises, isometric or isometric exercises or acute exercises or exercise, acute or exercises, acute or exercise training or exercise trainings or training, exercise or trainings, exercise or physical activity or activities, physical or activity, physical or physical activities).ab,ti,kw. | 418,654 |
| 3 | (exercise therapy or rehabilitation exercise or exercise, rehabilitation or exercises, rehabilitation or rehabilitation exercises or therapy, exercise or exercise therapies or therapies, exercise or remedial exercise or exercise, remedial or exercises, remedial or remedial exercises).ab,ti,kw. | 12,450 |
| 4 | Asthma/ | 148,727 |
| 5 | (asthma or asthmas or asthma, bronchial or bronchial asthma).ab,ti,kw. | 190,156 |
| 6 | Randomized Controlled Trial/ | 659,726 |
| 7 | randomized controlled trial.ab,ti,kw. | 148,618 |
| 8 | Controlled Clinical Trial/ | 95,745 |
| 9 | controlled clinical trial.ab,ti,kw. | 23,318 |
| 10 | 1 or 2 or 3 | 502,087 |
| 11 | 4 or 5 | 217,780 |
| 12 | 6 or 7 or 8 or 9 | 815,791 |
| 13 | 10 and 11 and 12 | 894 |

|  | | |
| --- | --- | --- |
| **Database 2: PubMed** | | |
| **No.** | **Search Strategy** | **Results** |
| #1 | "Exercise"[Mesh] OR "Exercise Therapy"[Mesh] | 326,842 |
| #2 | Exercise Prescription[Title/Abstract] OR Rehabilitation Exercise[Title/Abstract] OR Exercise, Rehabilitation[Title/Abstract] OR Exercises, Rehabilitation[Title/Abstract] OR Rehabilitation Exercises[Title/Abstract] OR Remedial Exercise[Title/Abstract] OR Exercise, Remedial[Title/Abstract] OR Exercises, Remedial[Title/Abstract] OR Remedial Exercises[Title/Abstract] OR Therapy, Exercise[Title/Abstract] OR Exercise Therapies[Title/Abstract] OR Therapies, Exercise[Title/Abstract] OR Physical Activity Intervention[Title/Abstract] | 10,731 |
| #3 | Exercises[Title/Abstract] OR Exercise, Physical[Title/Abstract] OR Exercises, Physical[Title/Abstract] OR Physical Exercise[Title/Abstract] OR Physical Exercises[Title/Abstract] OR Exercise, Isometric[Title/Abstract] OR Exercises, Isometric[Title/Abstract] OR Isometric Exercises[Title/Abstract] OR Isometric Exercise[Title/Abstract] OR Exercise, Aerobic[Title/Abstract] OR Aerobic Exercise[Title/Abstract] OR Aerobic Exercises[Title/Abstract] OR Exercises, Aerobic[Title/Abstract] OR Exercise Training[Title/Abstract] OR Exercise Trainings[Title/Abstract] OR Training, Exercise[Title/Abstract] OR Trainings, Exercise[Title/Abstract] OR Physical Activity[Title/Abstract] OR Activities, Physical[Title/Abstract] OR Activity, Physical[Title/Abstract] OR Physical Activities[Title/Abstract] OR Active Breaks[Title/Abstract] OR Activity Breaks[Title/Abstract] OR Acute Exercise[Title/Abstract] OR Acute Exercises[Title/Abstract] OR Exercise, Acute[Title/Abstract] OR Exercises, Acute[Title/Abstract] | 367,847 |
| #4 | #1 OR #2 OR #3 | 535,296 |
| #5 | "Asthma"[Mesh] | 151,746 |
| #6 | Asthmas[Title/Abstract] OR Asthma, Bronchial[Title/Abstract] OR Bronchial Asthma[Title/Abstract] | 20,774 |
| #9 | "Randomized Controlled Trial" [Publication Type] OR "Randomized Controlled Trials as Topic"[Mesh] OR "Controlled Clinical Trial" [Publication Type] | 941,842 |
| #10 | Clinical Trials, Randomized[Title/Abstract] OR Trials, Randomized Clinical[Title/Abstract] OR Controlled Clinical Trials, Randomized[Title/Abstract] | 257 |
| #11 | #5 OR #6 | 156,456 |
| #12 | #9 OR #10 | 942,027 |
| #13 | #4 AND #11 AND #12 | 345 |

|  | | |
| --- | --- | --- |
| **Database 3: Cochrane Library** | | |
| **No.** | **Search Strategy** | **Results** |
| #1 | MeSH descriptor: [Exercise] explode all trees | 42,845 |
| #2 | (Exercises OR Exercise, Physical OR Physical Exercises OR Physical Exercise OR Exercises, Physical OR Physical Activity OR Physical Activities OR Activity, Physical OR Activities, Physical OR Trainings, Exercise OR Training, Exercise OR Exercise Training OR Exercise Trainings OR Exercises, Isometric OR Exercise, Isometric OR Isometric Exercises OR Isometric Exercise OR Aerobic Exercises OR Aerobic Exercise OR Exercise, Aerobic OR Exercises, Aerobic OR Acute Exercises OR Acute Exercise OR Exercises, Acute OR Exercise, Acute OR Activity Breaks OR Active Breaks):ti,ab,kw | 222,502 |
| #3 | MeSH descriptor: [Exercise Therapy] explode all trees | 24,689 |
| #4 | (Physical Activity Intervention OR Remedial Exercise OR Exercise, Rehabilitation OR Rehabilitation Exercises OR Remedial Exercises OR Exercises, Remedial OR Exercise Therapies OR Exercise, Remedial OR Rehabilitation Exercise OR Exercise Prescription OR Therapy, Exercise OR Exercises, Rehabilitation OR Therapies, Exercise):ti,ab,kw | 130,331 |
| #5 | #1 OR #2 OR #3 OR #4 | 226,721 |
| #6 | MeSH descriptor: [Asthma] explode all trees | 14,794 |
| #7 | (Asthma, Bronchial OR Bronchial Asthma OR Asthmas):ti,ab,kw | 37,349 |
| #8 | #6 OR #7 | 37,358 |
| #9 | MeSH descriptor: [Randomized Controlled Trial] explode all trees | 34 |
| #10 | MeSH descriptor: [Controlled Clinical Trial] explode all trees | 37 |
| #11 | (Controlled Clinical Trials, Nonrandomized OR Controlled Clinical Trials, Non Randomized OR Controlled Clinical Trials, Non-Randomized OR Controlled Clinical Trials, Randomized):ti,ab,kw | 726,529 |
| #12 | (Veterinary Randomized Controlled Trial OR Non Randomized Controlled Trials as Topic):ti,ab,kw | 20,112 |
| #13 | #9 OR #10 OR #11 OR #12 | 729,627 |
| #14 | #13 AND #8 AND #5 | 826 |

| **Database 4: Embase** | | |
| --- | --- | --- |
| **No.** | **Search Strategy** | **Results** |
| #1 | 'exercise'/exp | 583,618 |
| #2 | 'biometric exercise':ti,ab,kw OR 'effort':ti,ab,kw OR 'exercise capacity':ti,ab,kw OR 'exercise performance':ti,ab,kw OR 'exercise training':ti,ab,kw OR 'exertion':ti,ab,kw OR 'fitness training':ti,ab,kw OR 'fitness workout':ti,ab,kw OR 'physical conditioning, human':ti,ab,kw OR 'physical effort':ti,ab,kw OR 'physical exercise':ti,ab,kw OR 'physical exertion':ti,ab,kw OR 'physical work-out':ti,ab,kw OR 'physical workout':ti,ab,kw OR 'exercise':ti,ab,kw | 806,178 |
| #3 | 'kinesiotherapy'/exp |  |
| #4 | 'corrective exercise':ti,ab,kw OR 'exercise movement techniques':ti,ab,kw OR 'exercise therapy':ti,ab,kw OR 'exercise treatment':ti,ab,kw OR 'kinesiotherapeutic intervention':ti,ab,kw OR 'kinesiotherapeutic method':ti,ab,kw OR 'kinesiotherapeutic procedure':ti,ab,kw OR 'kinesiotherapeutic technique':ti,ab,kw OR 'kinesiotherapeutical treatment':ti,ab,kw OR 'kinesiotherapeutic exercises':ti,ab,kw OR 'kinesiotherapeutic intervention':ti,ab,kw OR 'kinesitherapeutic method':ti,ab,kw OR 'kinesitherapeutic methodology':ti,ab,kw OR 'kinesitherapeutic procedure':ti,ab,kw OR 'kinesitherapeutic technique':ti,ab,kw OR 'kinesitherapeutic treatment':ti,ab,kw OR 'kinesitherapeutical treatment':ti,ab,kw OR 'kinesitherapy':ti,ab,kw OR 'sktm (specialized kinesitherapeutic methodology)':ti,ab,kw OR 'specialised kinesitherapeutic methodology':ti,ab,kw OR 'specialized kinesitherapeutic methodology':ti,ab,kw OR 'therapeutic exercise':ti,ab,kw OR 'therapy, exercise':ti,ab,kw OR 'treatment, exercise':ti,ab,kw OR 'kinesiotherapy':ti,ab,kw | 19,391 |
| #5 | #1 OR #2 OR #3 OR #4 | 1,050,223 |
| #6 | 'asthma'/exp | 366,886 |
| #7 | 'asthma bronchiale':ti,ab,kw OR 'asthma pulmonale':ti,ab,kw OR 'asthma, bronchial':ti,ab,kw OR 'asthmatic':ti,ab,kw OR 'asthmatic subject':ti,ab,kw OR 'bronchial asthma':ti,ab,kw OR 'bronchus asthma':ti,ab,kw OR 'chronic asthma':ti,ab,kw OR 'lung allergy':ti,ab,kw OR 'asthma':ti,ab,kw | 315,690 |
| #8 | 'randomized controlled trial'/exp | 1,204,896 |
| #9 | 'controlled trial, randomized':ti,ab,kw OR 'randomised controlled study':ti,ab,kw OR 'randomised controlled trial':ti,ab,kw OR 'randomized controlled study':ti,ab,kw OR 'trial, randomized controlled':ti,ab,kw OR 'randomized controlled trial':ti,ab,kw | 325,196 |
| #10 | #6 OR #7 | 411,468 |
| #11 | 'controlled clinical trial'/exp | 1,396,484 |
| #12 | 'clinical trial, controlled':ti,ab,kw OR 'controlled clinical comparison':ti,ab,kw OR 'controlled clinical drug trial':ti,ab,kw OR 'controlled clinical experiment':ti,ab,kw OR 'controlled clinical study':ti,ab,kw OR 'controlled clinical test':ti,ab,kw OR 'controlled clinical trial':ti,ab,kw | 46,181 |
| #13 | #8 OR #9 OR #11 OR #12 | 1,457,133 |
| #14 | #5 AND #10 AND #13 | 1,720 |

| **Database 5: Web of Science Core Collection** | | |
| --- | --- | --- |
| **No.** | **Search Strategy** | **Results** |
| #1 | (TS=(Exercise)) OR TS=(Exercise Therapy) | 689,724 |
| #2 | TI=(Exercises OR Exercise, Physical OR Exercises, Physical OR Physical Exercise OR Physical Exercises OR Exercise, Isometric OR Exercises, Isometric OR Isometric Exercises OR Isometric Exercise OR Exercise, Aerobic OR Aerobic Exercise OR Aerobic Exercises OR Exercises, Aerobic OR Exercise Training OR Exercise Trainings OR Training, Exercise OR Trainings, Exercise OR Physical Activity OR Activities, Physical OR Activity, Physical OR Physical Activities OR Active Breaks OR Activity Breaks OR Acute Exercise OR Acute Exercises OR Exercise, Acute OR Exercises, Acute) OR AB=(Exercises OR Exercise, Physical OR Exercises, Physical OR Physical Exercise OR Physical Exercises OR Exercise, Isometric OR Exercises, Isometric OR Isometric Exercises OR Isometric Exercise OR Exercise, Aerobic OR Aerobic Exercise OR Aerobic Exercises OR Exercises, Aerobic OR Exercise Training OR Exercise Trainings OR Training, Exercise OR Trainings, Exercise OR Physical Activity OR Activities, Physical OR Activity, Physical OR Physical Activities OR Active Breaks OR Activity Breaks OR Acute Exercise OR Acute Exercises OR Exercise, Acute OR Exercises, Acute) | 950,174 |
| #3 | TI=(Exercise Prescription OR Rehabilitation Exercise OR Exercise, Rehabilitation OR Exercises, Rehabilitation OR Rehabilitation Exercises OR Remedial Exercise OR Exercise, Remedial OR Exercises, Remedial OR Remedial Exercises OR Therapy, Exercise OR Exercise Therapies OR Therapies, Exercise OR Physical Activity Intervention) OR AB=(Exercise Prescription OR Rehabilitation Exercise OR Exercise, Rehabilitation OR Exercises, Rehabilitation OR Rehabilitation Exercises OR Remedial Exercise OR Exercise, Remedial OR Exercises, Remedial OR Remedial Exercises OR Therapy, Exercise OR Exercise Therapies OR Therapies, Exercise OR Physical Activity Intervention) | 146,044 |
| #4 | TS=(Asthma) | 244,504 |
| #5 | TI=(Asthmas OR Asthma, Bronchial OR Bronchial Asthma) OR AB=(Asthmas OR Asthma, Bronchial OR Bronchial Asthma) | 22,493 |
| #6 | ALL=(Randomized Controlled Trial OR Randomized Controlled Trials as Topic OR Controlled Clinical Trial) | 840,753 |
| #7 | #1 OR #2 OR #3 | 1,051,685 |
| #8 | #5 OR #4 | 244,518 |
| #9 | #7 AND #8 AND #6 | 814 |

# Supplementary Table 2: Characteristics of the included studies.

| **Author (Year)** | **Participant** | | **Severity** | **Interventions** | **Control** | **Outcome** | |
| --- | --- | --- | --- | --- | --- | --- | --- |
|  | **IG** | **CG** |  |  |  | **PAQLQ** | **Spirometry** |
| M. Arandelović (2007) | N=45; Age: 33.07±9.81; 24.4% female | N=20; Age: 33.55±10.88; 33.3% female | Mild | Swimming + low-dose medication  Duration: 6mth  Frequency: 2/wk  Session: 1h | Conventional drug treatment | NA | FEV₁ L  PEF L/s |
| S. Basaran (2006) | N=30; Age: 10.35±2.2; 35% female; BMI: 19.0(3.8) | N=28; Age: 10.45(2.1); 35% female; BMI: 17.7(3.2) | Mild to moderate | Submaximal intensity aerobic training  Duration: 8wk  Frequency: 3/wk  Session: 55min | Usual care | PAQLQ | PEF % predicted |
| J. S. Wang (2009) | N=15; Age: 10; 50% female; BMI: 20.6 | N=15; Age: 10; 50% female; BMI: 19.5 | Not reported | Swimming, Water breathing exercises  Duration: 6wk  Frequency: 3/wk  Session: 50min | Usual care | NA | FEV₁ % predicted |
| P. Yadav (2021) | N=70; Age: 11.74±1.56; 35% female; BMI: 18.46±2.68 | N=70; Age: 12.11±1.74; 38% female; BMI: 18.6±3.13 | Not reported | Yoga  Duration: 12wk  Frequency: 7/wk  Session: 45min | Usual care | PAQLQ | FEV₁ % predicted |
| A.M.Yousef (2022) | N=20; Age: 10.2±1.39; 35% female; BMI: 17.07±1.25 | N=20; Age: 10.47±1.25; 45% female; BMI: 17.33±1.16 | Moderate | Aerobic training on treadmill, Breathing exercises  Duration: 12wk  Frequency: 5/wk  Session: 30min | Usual care | PAQLQ | FEV₁ % predicted |
| Jia Li (2016) | MCT: N=14  Age: 11.7±2.0  35.7% female  BMI: 19.3±2.9 | N=12; Age: 10.8±1.3; 33.3% female; BMI: 18.8±2.9 | Not reported | MCT: Pedal power wagon  Duration: 8wk  Frequency: 4/wk  Session: 8min | Usual care | NA | FEV₁ % predicted  FVC % predicted  PEF % predicted |
|  | HIIT: N=15  Age: 12.5±1.6  40.0% female  BMI: 18.5±2.7 |  |  | HIIT: Pedal power wagon  Duration: 8wk  Frequency: 3/wk  Session: 40min |  |  |  |
| Guang xin Li (2016) | N=23; Age: 11.9(2.3); BMI: 20.8(2.7) | N=23; Age: 12.5(3.0); BMI: 19.3(3.1) | Mild | Pedal power wagon  Duration: 8wk  Frequency: 3/wk  Session: 32–36min | Usual care | NA | FEV₁ % predicted  FVC % predicted  PEF % predicted |
| R. Vempati (2009) | N=29; Age: 33.5±11.4; 45% male; BMI: 23.4±4.3 | N=28; Age: 33.4±11.5; 71% male; BMI: 22.6±4.0 | Mild to moderate | Yoga, Breathing Exercises  Duration: 8wk  Frequency: 7/wk  Session: 60min | Usual care | NA | FEV₁ % predicted  FVC % predicted |
| B. S. Shaw (2011) | AE: N=22  Age: 21.95±3.87  Stature: 170.88±9.17cm  Body mass: 78.40±17.80kg | N=22; Age: 21.90±3.89; Body mass: 76.05±13.95kg; Stature: 169.37±10.68cm | Moderate | AE: Aerobic exercise  Duration: 8wk  Frequency: 3/wk  Session: 30min | Non-intervention | NA | FEV₁ L  FVC L  PEF L/s |
|  | DR: N=22  Age: 21.93±3.95  Stature: 168.66±7.53cm  Body mass: 75.18±12.66kg |  |  | DR: Diaphragmatic breathing  Duration: 8wk  Frequency: 3/wk |  |  |  |
|  | CE: N=22  Age: 22.00±3.95  Stature: 172.00±10.14cm  Body mass: 77.08±9.74kg |  |  | CE: Walking and Jogging  Duration: 8wk  Frequency: 3/wk  Session: 15min |  |  |  |
| R. Farid (2005) | N=18; Age: 27; 44% male | N=18; Age: 29; 44% male | Not reported | Cardio training  Duration: 8wk  Frequency: 3/wk  Session: 20min | Non-intervention | NA | FEV₁ % predicted  FVC % predicted |
| N. H. van Veldhoven (2001) | N=23; Age: 10.5±1.2; Body mass: 39.4±11.8kg; Stature: 144.6±10.4cm | N=24; Age: 10.7±1.2; Body mass: 38.4±9.1kg; Stature: 144.8±8.4cm | Not reported | Mixed fitness activities  Duration: 3mth  Frequency: 3/wk  Session: 1h20min | Non-intervention | NA | FEV₁ L  FVC L  FEV₁ % predicted  PEF L/s |
| P. Latorre-Román (2014) | N=58; Age: 11.55±1.01; BMI: 19.69±3.20 | N=47; Age: 11.51±1.42; BMI: 21.39±4.78 | Not reported | Alternating high and low intensity training  Duration: 12wk  Frequency: 3/wk  Session: 60min | Non-intervention | PAQLQ | FVC L  PEF L/s |
| F.P. Counil (2003) | N=7; Age: 14±0.6 | N=7; Age: 13.9±0.8 | Mild to moderate | Cycling training  Duration: 6wk  Frequency: 3/wk  Session: 45min | Usual care | NA | FEV₁ % predicted |
| L.M. Cochrane (1990) | N=18; Age: 27±7 | N=18; Age: 28±8 | Mild to moderate | Various submaximal intensity aerobic exercises  Duration: 3mth  Frequency: 3/wk  Session: 30min | Non-intervention | NA | FEV₁ % predicted  FEV₁ L |
| L. B. Andrade (2014) | N=10; Age: 11.7(2.3); 40% female; BMI: 20.9±6.1 | N=17; Age: 11.4(2.3); 47.1% female; BMI: 18.7±3.9 | Moderate | Treadmill aerobic training  Duration: 6wk  Frequency: 3/wk  Session: 20–30min | Usual care | PAQLQ | / |
| W. K. Abdelbasset (2018) | N=19; Age: 9.84±1.76; 36.8% female; BMI: 21.3±3.02 | N=19; Age: 10.04±1.52; 42.1% female; BMI: 22.13±4.1 | Moderate | Moderate-intensity aerobic exercise + asthma medication  Duration: 10wk  Frequency: 3/wk  Session: 40min | Conventional drug treatment | PAQLQ | FEV₁ % predicted |
| A. Refaat (2015) | N=38; Age: 35.8±1.7; 55.3% female; BMI: 23±1.8 | N=30; Age: 38±5.3; 53.3% female; BMI: 22±0.7 | Not reported | Mixed endurance training  Duration: 6wk  Frequency: 3/wk  Session: 30–40min | Usual care | NA | FEV₁ L |
| C. Carew (2017) | Swimming: N=9  Age: 13.3±1.9  44.4% female  Body mass: 57.9±14.3kg  Stature: 160.2±10.7cm | N=10; Age: 12.0±3.1; 66.7% female; Body mass: 50.0±16.4kg; Stature: 155.9±15.9cm | Mild to moderate | Swimming  Duration: 6wk  Frequency: 1/wk  Session: 40min | Non-intervention | NA | FEV₁ % predicted  FVC % predicted  PEF % predicted |
|  | Football: N=9  Age: 13.2±1.6  22.2% female  Body mass: 58.2±12.3kg  Stature: 163.4±10.5cm |  |  | Football  Duration: 6wk  Frequency: 1/wk  Session: 40min |  |  |  |
|  | Basketball: N=11  Age: 13.5±1.8  18.2% female  Body mass: 62.3±13.9kg  Stature: 166.9±13.0cm |  |  | Basketball  Duration: 6wk  Frequency: 1/wk  Session: 40min |  |  |  |
| Micozzi. S (2025) | N=26; Age: 35.7±8.8; 11.5% male; BMI: 25.5±5.1 | N=26; Age: 36.7±8.9; 42.3% male; BMI: 24.8±3.3 | Mild to moderate | Walk  Duration: 3mth  Frequency: 7/wk  Session: 7000 steps | Non-intervention | NA | FEV₁ L |
| Sangeethalaxmi, MJ (2023) | N=30; Age: 24.2±2.63 | N=30; Age: 22.97±2.54 | Mild to moderate | Yoga  Duration: 3mth  Frequency: 7/wk | Usual care | NA | FEV₁ L  FVC L  PEF L/s |
| Saravanakumar, T (2026) | N=45; Age: 39.15±3.73; BMI: 25.65±4.37; 100% male | N=44; Age: 37.7±4.14; BMI: 27.14±4.88; 100% male | Mild | Yoga  Duration: 3mth  Frequency: 4/wk  Session: 1h | Usual care | NA | FEV₁ L  FVC L |
| Sodhi, C (2009) | N=34; Age: 38.77±9.92 | N=37; Age: 35.55±10.62 | Mild to moderate | Yoga  Duration: 8wk  Frequency: 1/wk  Session: 45min | Conventional drug treatment | NA | FEV₁ % predicted  FVC % predicted  PEF % predicted |

*Note.* IG, intervention group; CG, control group; N, sample size; BMI, body mass index (kg/m²); PAQLQ, Pediatric Asthma Quality of Life Questionnaire; FEV_1_, forced expiratory volume in one second; FEV_1_% predicted, FEV_1_ as a percentage of predicted value; FVC, forced vital capacity; FVC% predicted, FVC as a percentage of predicted value; PEF, peak expiratory flow; PEF% predicted, PEF as a percentage of predicted value; L, liters; L/s, liters per second; wk, weeks; mth, months; min, minutes; h, hours; MCT, moderate-intensity continuous training; HIIT, high-intensity interval training; AE, aerobic exercise; DR, diaphragmatic respiration; CE, combined exercise; NA, not available.

*Asthma severity was classified into four categories based on the original study reports: mild, mild-to-moderate, moderate, and not reported. Studies that did not specify severity were classified as “not reported.”*

*Multi-arm studies: Three studies (Carew 2017, Shaw 2011, and Jia Li 2016) reported multiple intervention arms with a single shared control group. Following the Cochrane Handbook (Section 6.5.2.10) recommendation, the control group sample size was proportionally divided across intervention arms to create independent comparison units. In this table, control group data are presented in the first row of each multi-arm study, and subsequent rows for additional arms share the same control.*

*“X/wk” denotes the number of intervention sessions per week (e.g., 3/wk = three sessions per week).*

**Supplementary Table 3. Random-effects meta-regression of intervention-level covariates on FEV_1_% predicted (k = 13 studies, 16 effect sizes).**

| **Model** | **Variable** | ***β*** | **95% CI** | **P** | **Residual I²** | **R²** |
| --- | --- | --- | --- | --- | --- | --- |
| Univariable 1 | Duration (weeks) | −0.030 | −1.985, 1.925 | 0.974 | 86.84% | 0.00% |
| Univariable 2 | Frequency (sessions/week) | 0.548 | −1.832, 3.074 | 0.647 | 88.19% | 0.00% |
| Univariable 3 | Session duration (min) | 0.043 | −0.040, 0.126 | 0.281 | 88.27% | 0.91% |
| Multivariable | Duration (weeks) | −0.062 | −2.121, 1.998 | 0.950 | 87.53% | 0.00% |
|  | Aerobic type | 0.885 | −8.525, 10.296 | 0.842 |  |  |

*Note.* β, regression coefficient; 95% CI, 95% confidence interval; Residual *I*², residual between-study heterogeneity after accounting for the covariate(s); R², proportion of between-study variance explained by the model; FEV_1_% predicted, forced expiratory volume in one second as a percentage of predicted value. All models used random-effects meta-regression with the restricted maximum likelihood (REML) estimator. “Aerobic type” is a binary indicator (aerobic vs non-aerobic).

**Supplementary Table 4. Random-effects meta-regression of intervention-level covariates on FVC% predicted (k = 11 studies, 14 effect sizes).**

| **Model** | **Variable** | ***β*** | **95% CI** | **P** | **Residual I²** | **R²** |
| --- | --- | --- | --- | --- | --- | --- |
| Univariable 1 | Duration (weeks) | 0.341 | −3.089, 3.772 | 0.831 | 87.97% | 0.00% |
| Univariable 2 | Frequency (sessions/week) | −0.572 | −4.326, 3.181 | 0.744 | 92.69% | 0.00% |
| Univariable 3 | Session duration (min) | −0.017 | −0.129, 0.094 | 0.74 | 92.61% | 0.00% |
| Multivariable | Duration (weeks) | 0.041 | −3.534, 3.617 | 0.98 | 87.23% | 0.00% |
|  | Aerobic type | −5.371 | −17.841, 7.097 | 0.36 |  |  |

*Note.* β, regression coefficient; 95% CI, 95% confidence interval; Residual *I*², residual between-study heterogeneity after accounting for the covariate(s); R², proportion of between-study variance explained by the model; FVC% predicted, forced vital capacity as a percentage of predicted value. All models used random-effects meta-regression with the restricted maximum likelihood (REML) estimator. “Aerobic type” is a binary indicator (aerobic vs non-aerobic).

# Supplementary Table 5. Subgroup analyses for FEV_1_% predicted and FVC% predicted.

| **Outcome** | **Subgroup variable** | **Subgroup** | **k** | **MD** | **95% CI** | **P (Overall Effect)** | **I², %** | **Qb** | **P (Group difference)** |
| --- | --- | --- | --- | --- | --- | --- | --- | --- | --- |
| **FEV₁% predicted** | **Age** | **<18 years** | **13** | **3.81** | **[1.30, 6.31]** | **<0.001** | **60.07** | **3.60** | **0.06** |
|  |  | **≥18 years** | **3** | **17.48** | **[3.59, 31.37]** | **0.013** | **91.46** |  |  |
|  | **Exercise type** | **Aerobic** | **7** | **5.31** | **[−2.82, 13.44]** | **0.20** | **91.23** | **0.25** | **0.62** |
|  |  | **Non-aerobic** | **8** | **7.40** | **[6.54, 8.26]** | **<0.001** | **0.00** |  |  |
|  | **Intervention duration** | **>8 weeks** | **5** | **7.42** | **[6.55, 8.30]** | **<0.001** | **0.00** | **0.13** | **0.72** |
|  |  | **≤8 weeks** | **11** | **6.35** | **[0.53, 12.18]** | **0.033** | **85.10** |  |  |
|  | **Weekly frequency** | **>3 sessions/week** | **3** | **7.85** | **[−1.72, 17.41]** | **0.10** | **81.77** | **0.23** | **0.63** |
|  |  | **≤3 sessions/week** | **13** | **5.28** | **[0.81, 9.76]** | **0.020** | **89.30** |  |  |
|  | **Session duration** | **≥45 min** | **6** | **5.12** | **[0.42, 9.81]** | **0.033** | **77.05** | **0.07** | **0.80** |
|  |  | **<45 min** | **10** | **6.09** | **[0.28, 11.90]** | **0.040** | **88.63** |  |  |
| **FVC% predicted** | **Age** | **<18 years** | **11** | **3.58** | **[1.93, 5.22]** | **<0.001** | **18.28** | **0.13** | **0.72** |
|  |  | **≥18 years** | **3** | **7.69** | **[−14.84, 30.22]** | **0.50** | **97.31** |  |  |
|  | **Exercise type** | **Aerobic** | **6** | **5.52** | **[−4.49, 15.52]** | **0.28** | **91.52** | **0.40** | **0.53** |
|  |  | **Non-aerobic** | **8** | **2.11** | **[−1.36, 5.58]** | **0.23** | **73.24** |  |  |
|  | **Intervention duration** | **>8 weeks** | **3** | **4.47** | **[3.75, 5.20]** | **<0.001** | **0.00** | **0.30** | **0.58** |
|  |  | **≤8 weeks** | **11** | **2.69** | **[−3.60, 8.98]** | **0.40** | **85.06** |  |  |
|  | **Weekly frequency** | **>3 sessions/week** | **3** | **−2.46** | **[−10.67, 5.76]** | **0.56** | **79.39** | **2.35** | **0.12** |
|  |  | **≤3 sessions/week** | **10** | **5.23** | **[−0.15, 10.61]** | **0.06** | **90.29** |  |  |
|  | **Session duration** | **≥45 min** | **4** | **−0.06** | **[−6.39, 6.28]** | **0.99** | **82.31** | **1.28** | **0.26** |
|  |  | **<45 min** | **10** | **5.04** | **[−1.09, 11.17]** | **0.11** | **89.74** |  |  |

***Note.* k, number of effect sizes; MD, mean difference; 95% CI, 95% confidence interval; P (Overall Effect), P value for the pooled effect within the subgroup; *I*², inconsistency statistic indicating within-subgroup heterogeneity; *Q*_b_, Cochran’s Q statistic for between-subgroup difference; P (Group difference), P value for the between-subgroup comparison. All models used random-effects meta-analysis with the restricted maximum likelihood (REML) estimator. Non-aerobic exercise includes yoga, breathing exercises, ball sports (basketball, football), and mixed non-aerobic training. FEV_1_% predicted, forced expiratory volume in one second as a percentage of predicted value; FVC% predicted, forced vital capacity as a percentage of predicted value.**

# Supplementary Table 6. GRADE Summary of Findings for the primary and key secondary outcomes.

**Authors: Youjia Mao and Zijian Zhu**

**Question:** Physical activity compared to usual care for asthma

| **Certainty assessment** | | | | | | | **№ of patients** | | **Effect** | | **Certainty** | **Importance** |
| --- | --- | --- | --- | --- | --- | --- | --- | --- | --- | --- | --- | --- |
| **№ of studies** | **Study design** | **Risk of bias** | **Inconsistency** | **Indirectness** | **Imprecision** | **Other considerations** | **physical activity** | **usual care** | **Relative (95% CI)** | **Absolute (95% CI)** |  |  |
| **FEV₁% predicted** | | | | | | | | | | | | |
| 13 | randomised trials | serious^a^ | serious^b^ | not serious | not serious | none | 358 | 323 | - | MD **5.79 higher** (1.89 higher to 9.69 higher) | ⨁⨁◯◯ Low^a,b^ |  |
| **FVC% predicted** | | | | | | | | | | | | |
| 11 | randomised trials | serious^c^ | very serious^d^ | not serious | not serious | none | 340 | 302 | - | MD **3.41 higher** (1.32 lower to 8.14 higher) | ⨁◯◯◯ Very low^c,d^ |  |
| **PAQLQ** | | | | | | | | | | | | |
| 6 | randomised trials | serious^e^ | very serious^f^ | not serious | not serious | none | 207 | 201 | - | MD **1.13 higher** (0.45 higher to 1.81 higher) | ⨁◯◯◯ Very low^e,f^ |  |
| **PEF% predicted** | | | | | | | | | | | | |
| 7 | randomised trials | serious^g^ | very serious^h^ | not serious | serious^i^ | none | 207 | 170 | - | MD **5.24 higher** (0.9 lower to 11.37 higher) | ⨁◯◯◯ Very low^g,h,i^ |  |

***Note.* GRADE, Grading of Recommendations Assessment, Development and Evaluation; MD, mean difference; 95% CI, 95% confidence interval; FEV_1_% predicted, forced expiratory volume in one second as a percentage of predicted value; FVC% predicted, forced vital capacity as a percentage of predicted value; PAQLQ, Pediatric Asthma Quality of Life Questionnaire; PEF% predicted, peak expiratory flow as a percentage of predicted value. ⨁⨁⨁⨁ High certainty; ⨁⨁⨁◯ Moderate certainty; ⨁⨁◯◯ Low certainty; ⨁◯◯◯ Very low certainty.**

**Explanations**

1. **Only 2 of 13 studies (15.4%) were rated as low risk of bias overall; 8 studies had some concerns and 3 were rated as high risk. The proportion of low-risk studies fell below the 25% threshold, warranting a one-level downgrade.**
2. ***I*² = 89.16%, indicating very high statistical heterogeneity. However, subgroup analyses identified two consistent low-heterogeneity patterns: the non-aerobic exercise subgroup (*I*² = 0.00%, k = 8) and the long-duration subgroup (>8 weeks, *I*² = 0.00%, k = 5) both demonstrated homogeneous effects, suggesting that heterogeneity is partly attributable to exercise type and intervention duration. Leave-one-out analysis confirmed that effect estimates remained statistically significant across all iterations (MD range: 4.67–6.26, all P ≤ 0.010), indicating a consistent effect direction despite the high between-study variance. Downgraded by one level only, as heterogeneity sources were partially identified and the effect direction was consistent.**
3. **2 of 11 studies (18.2%) were rated as low risk, 5 had some concerns, and 4 were rated as high risk (36.4%). The high proportion of high-risk studies warrants a one-level downgrade.**
4. ***I*² = 92.13%, indicating extreme heterogeneity that exceeded the 90% threshold. Subgroup analyses did not identify clear heterogeneity sources. Downgraded by two levels.**
5. **2 of 6 studies (33.3%) were low risk, 2 had some concerns, and 2 were high risk (33.3%). The limited number of low-risk studies and the balanced but concerning distribution warrant a one-level downgrade.**
6. ***I*² = 96.14%, representing extreme and concerning heterogeneity that substantially exceeded the 90% threshold. This warrants a two-level downgrade for inconsistency.**
7. **Only 1 of 7 studies (14.3%) was low risk, 3 had some concerns, and 3 were high risk (42.9%). This is the lowest proportion of low-risk studies among all outcomes; risk of bias is a serious concern. Downgraded by one level.**
8. ***I*² = 90.95%, indicating very high heterogeneity that exceeded the 90% threshold. Downgraded by two levels.**
9. **The 95% CI [−0.90, 11.37] crosses the null value, and the wide confidence interval combined with a small number of studies (k = 7) limits precision. Downgraded by one level.**


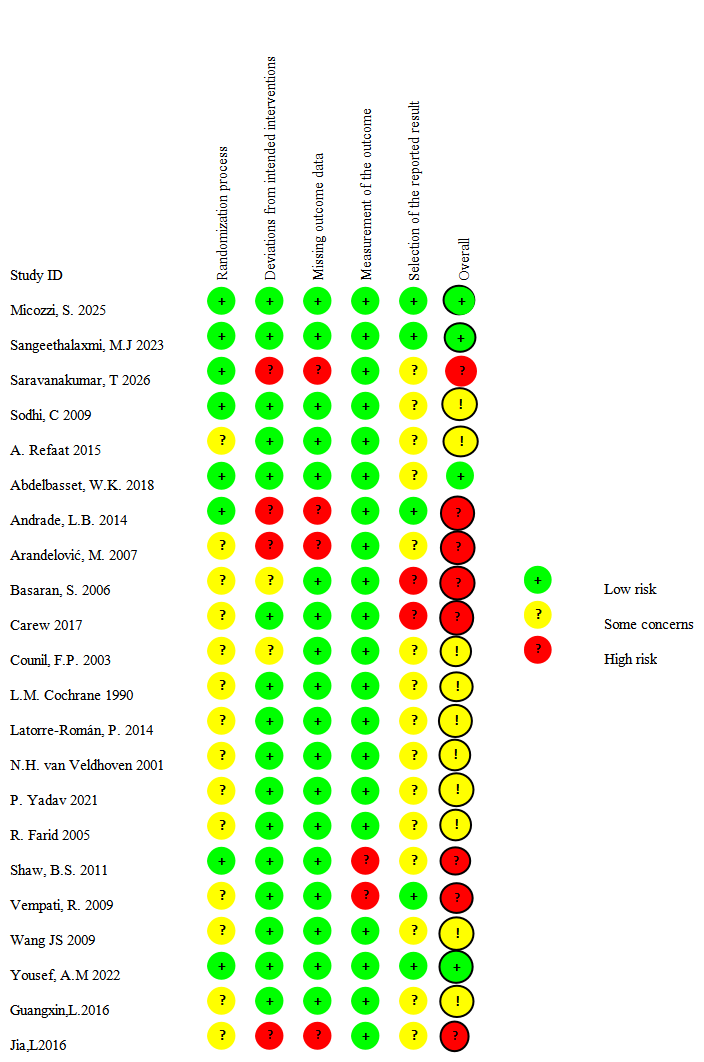


**Supplementary Figure 1. Risk-of-bias traffic light plot for the 22 included randomized controlled trials, assessed using the Cochrane Risk-of-Bias 2 (RoB 2) tool. Each row represents one study and each column corresponds to one of the five RoB 2 domains: (D1) bias arising from the randomization process; (D2) bias due to deviations from the intended interventions; (D3) bias due to missing outcome data; (D4) bias in measurement of the outcome; (D5) bias in selection of the reported result. Green, low risk of bias; yellow, some concerns; red, high risk of bias.**

**Supplementary Figure 2. Summary plot of the risk-of-bias assessment across the 22 included randomized controlled trials. Bars indicate the proportion of studies judged as low risk (green), some concerns (yellow), and high risk (red) for each of the five RoB 2 domains and the overall judgment. RoB 2, Cochrane Risk-of-Bias 2 tool.**

**
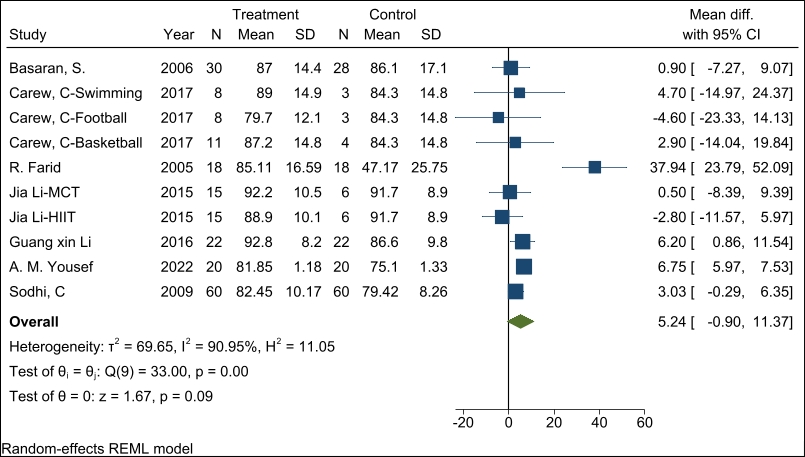
**

**Supplementary Figure 3. Forest plot of the random-effects meta-analysis of physical activity on PEF% predicted in patients with asthma. Effect estimates are expressed as mean differences with 95% confidence intervals. The pooled estimate was calculated using the restricted maximum likelihood (REML) estimator. Mean diff, mean difference; SD, standard deviation; 95% CI, 95% confidence interval; PEF% predicted, peak expiratory flow as a percentage of predicted value; REML, restricted maximum likelihood; MCT, moderate-intensity continuous training; HIIT, high-intensity interval training.**

**
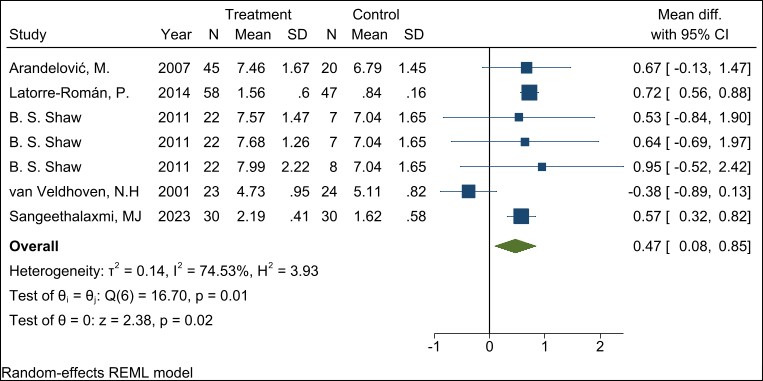
**

**Supplementary Figure 4. Forest plot of the random-effects meta-analysis of physical activity on PEF (L/s) in patients with asthma. Effect estimates are expressed as mean differences with 95% confidence intervals. The pooled estimate was calculated using the restricted maximum likelihood (REML) estimator. Mean diff, mean difference; SD, standard deviation; 95% CI, 95% confidence interval; PEF, peak expiratory flow; L/s, liters per second; REML, restricted maximum likelihood; AE, aerobic exercise; DR, diaphragmatic respiration; CE, combined exercise.**

**
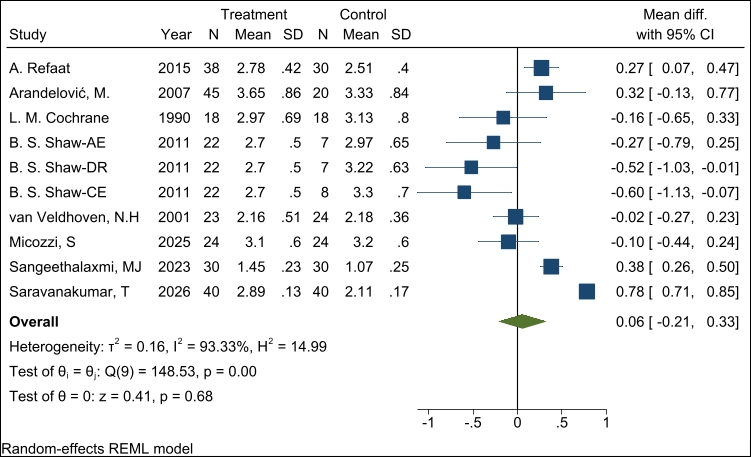
**

**Supplementary Figure 5. Forest plot of the random-effects meta-analysis of physical activity on FEV_1_ (L) in patients with asthma. Effect estimates are expressed as mean differences with 95% confidence intervals. The pooled estimate was calculated using the restricted maximum likelihood (REML) estimator. Mean diff, mean difference; SD, standard deviation; 95% CI, 95% confidence interval; FEV_1_, forced expiratory volume in one second; L, liters; REML, restricted maximum likelihood; AE, aerobic exercise; DR, diaphragmatic respiration; CE, combined exercise.**

**
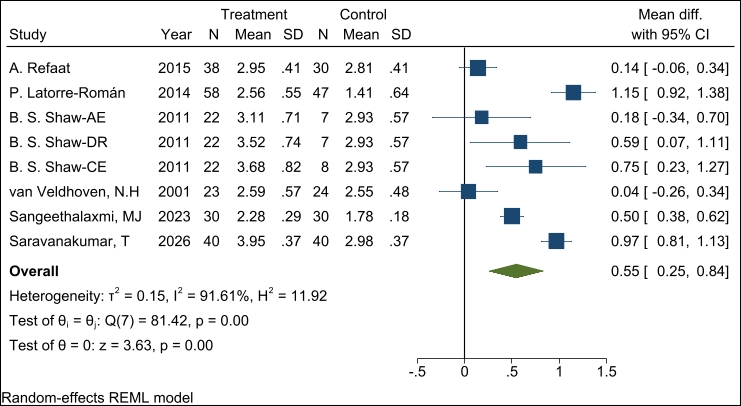
**

**Supplementary Figure 6. Forest plot of the random-effects meta-analysis of physical activity on FVC (L) in patients with asthma. Effect estimates are expressed as mean differences with 95% confidence intervals. The pooled estimate was calculated using the restricted maximum likelihood (REML) estimator. Mean diff, mean difference; SD, standard deviation; 95% CI, 95% confidence interval; FVC, forced vital capacity; L, liters; REML, restricted maximum likelihood; AE, aerobic exercise; DR, diaphragmatic respiration; CE, combined exercise.**

**
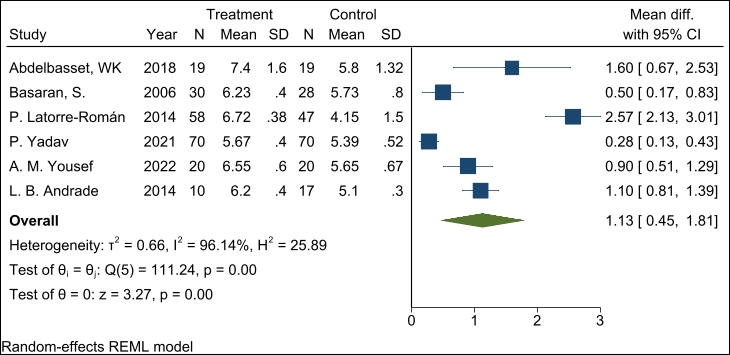
**

**Supplementary Figure 7. Forest plot of the random-effects meta-analysis of physical activity on the Pediatric Asthma Quality of Life Questionnaire (PAQLQ) total score in patients with asthma. Effect estimates are expressed as mean differences with 95% confidence intervals. The pooled estimate was calculated using the restricted maximum likelihood (REML) estimator. Mean diff, mean difference; SD, standard deviation; 95% CI, 95% confidence interval; PAQLQ, Pediatric Asthma Quality of Life Questionnaire; REML, restricted maximum likelihood.**

**
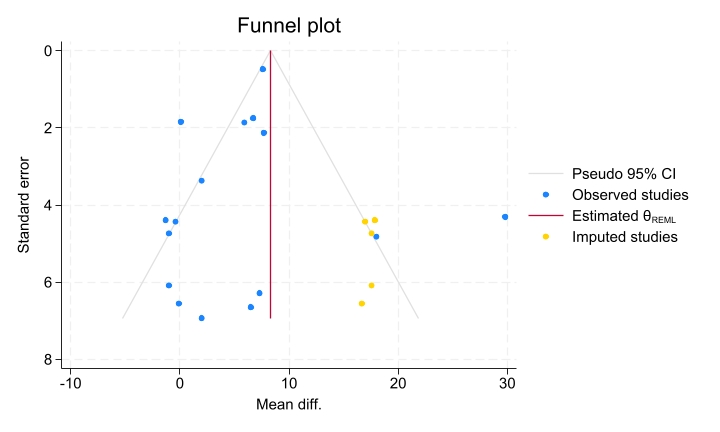
**

**Supplementary Figure 8. Contour-enhanced funnel plot with Duval and Tweedie trim-and-fill imputation for FEV_1_% predicted. Blue markers represent observed studies and yellow markers represent imputed studies. FEV_1_% predicted, forced expiratory volume in one second as a percentage of predicted value; SE, standard error.**

**
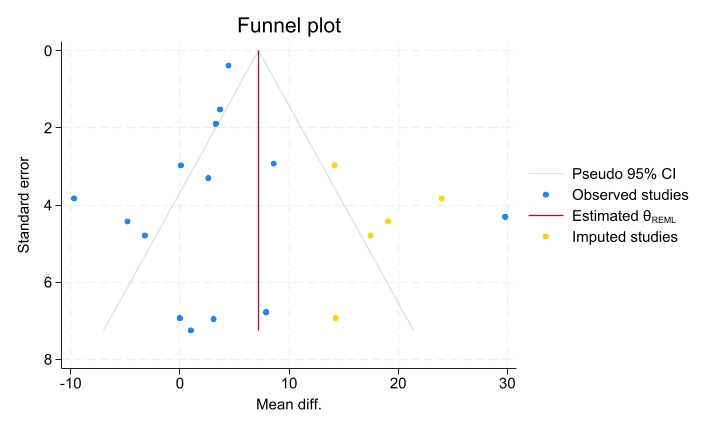
**

**Supplementary Figure 9. Contour-enhanced funnel plot with Duval and Tweedie trim-and-fill imputation for FVC% predicted. Blue markers represent observed studies and yellow markers represent imputed studies. FVC% predicted, forced vital capacity as a percentage of predicted value; SE, standard error.**

**Supplementary Figure 10.** Leave-one-out sensitivity analysis for the primary outcome FEV_1_% predicted. Each circle represents the recalculated pooled estimate after omitting the named study (or effect size for multi-arm comparisons), with the corresponding 95% confidence interval shown as horizontal lines. FEV_1_% predicted, forced expiratory volume in one second as a percentage of predicted value; 95% CI, 95% confidence interval.


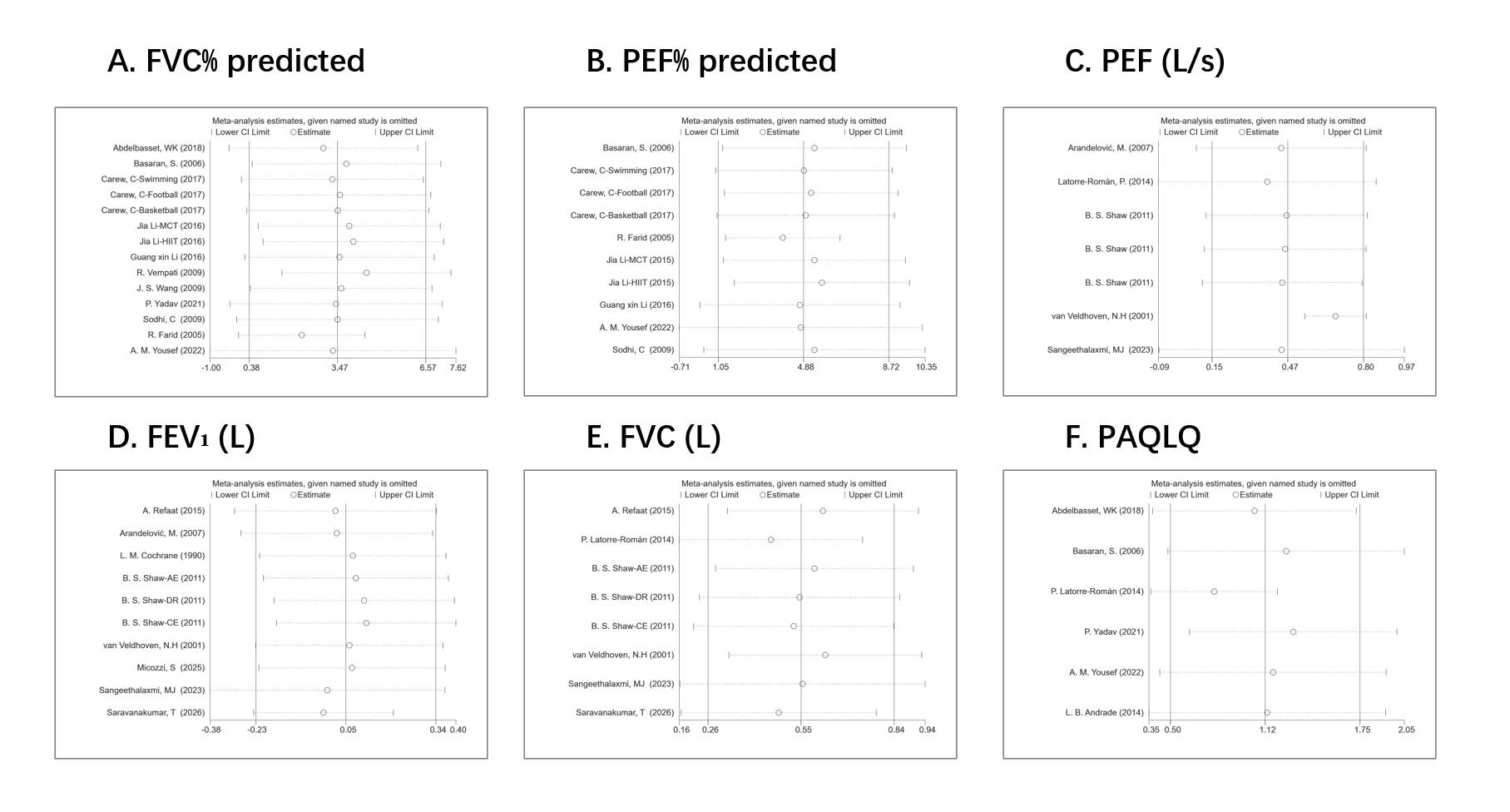


**Supplementary Figure 11.** Leave-one-out sensitivity analyses for the secondary outcomes. (A) FVC% predicted; (B) PEF% predicted; (C) PEF (L/s); (D) FEV_1_ (L); (E) FVC (L); (F) PAQLQ. Each circle represents the recalculated pooled estimate after omitting the named study (or effect size for multi-arm comparisons), with the corresponding 95% confidence interval shown as horizontal lines. FVC% predicted, forced vital capacity as a percentage of predicted value; PEF% predicted, peak expiratory flow as a percentage of predicted value; PEF, peak expiratory flow; L/s, liters per second; FEV_1_, forced expiratory volume in one second; L, liters; FVC, forced vital capacity; PAQLQ, Pediatric Asthma Quality of Life Questionnaire; 95% CI, 95% confidence interval.


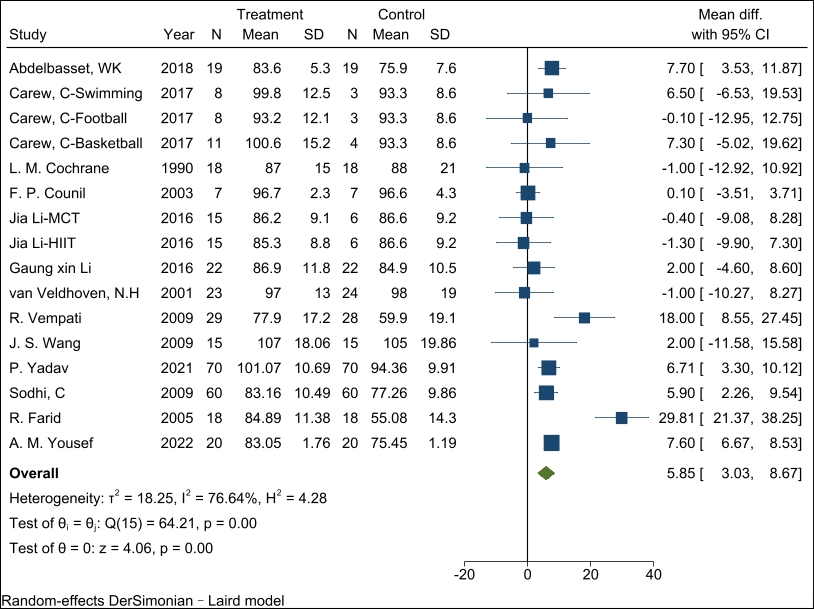


**Supplementary Figure 12.** Forest plot of the meta-analysis of physical activity on FEV_1_% predicted using the DerSimonian-Laird (DL) heterogeneity variance estimator, performed as a sensitivity analysis. Effect estimates are expressed as mean differences with 95% confidence intervals. Mean diff, mean difference; SD, standard deviation; 95% CI, 95% confidence interval; FEV_1_% predicted, forced expiratory volume in one second as a percentage of predicted value; DL, DerSimonian-Laird.


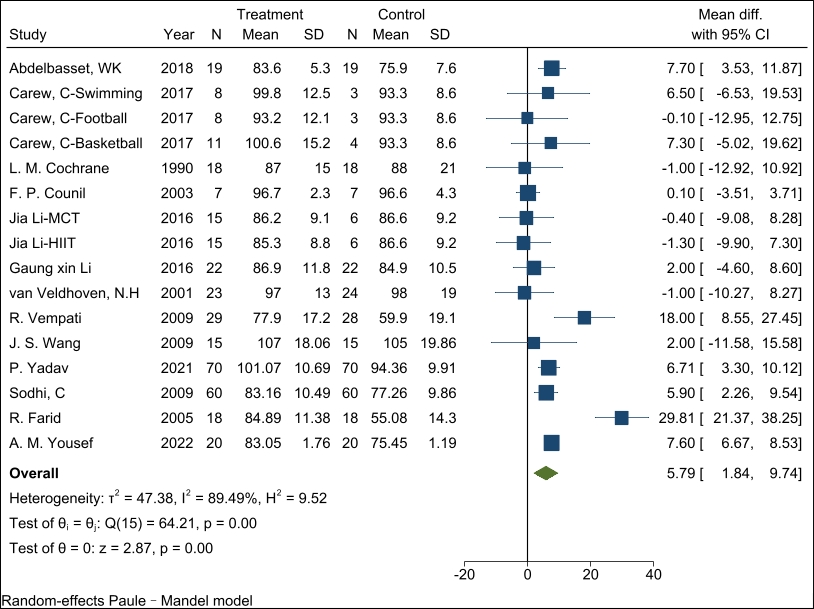


**Supplementary Figure 13.** Forest plot of the meta-analysis of physical activity on FEV_1_% predicted using the Paule-Mandel (PM) heterogeneity variance estimator, performed as a sensitivity analysis. Effect estimates are expressed as mean differences with 95% confidence intervals. Mean diff, mean difference; SD, standard deviation; 95% CI, 95% confidence interval; FEV_1_% predicted, forced expiratory volume in one second as a percentage of predicted value; PM, Paule-Mandel.


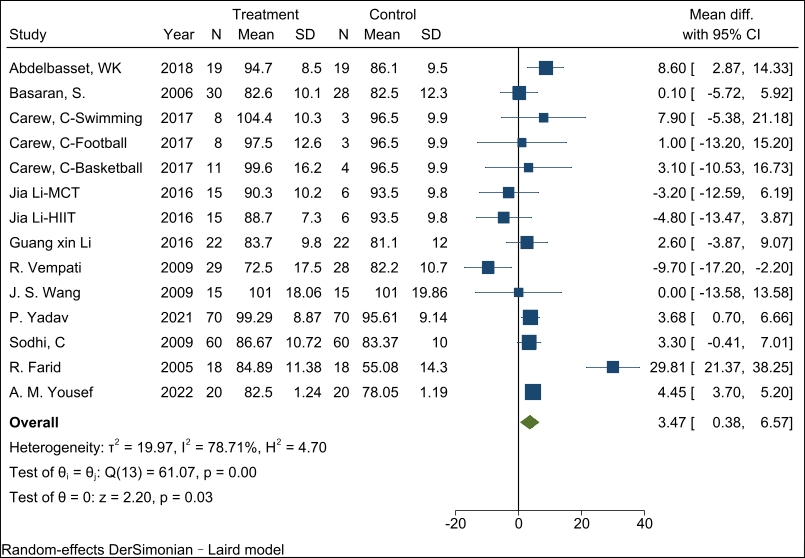


**Supplementary Figure 14.** Forest plot of the meta-analysis of physical activity on FVC% predicted using the DerSimonian-Laird (DL) heterogeneity variance estimator, performed as a sensitivity analysis. Effect estimates are expressed as mean differences with 95% confidence intervals. Mean diff, mean difference; SD, standard deviation; 95% CI, 95% confidence interval; FVC% predicted, forced vital capacity as a percentage of predicted value; DL, DerSimonian-Laird.


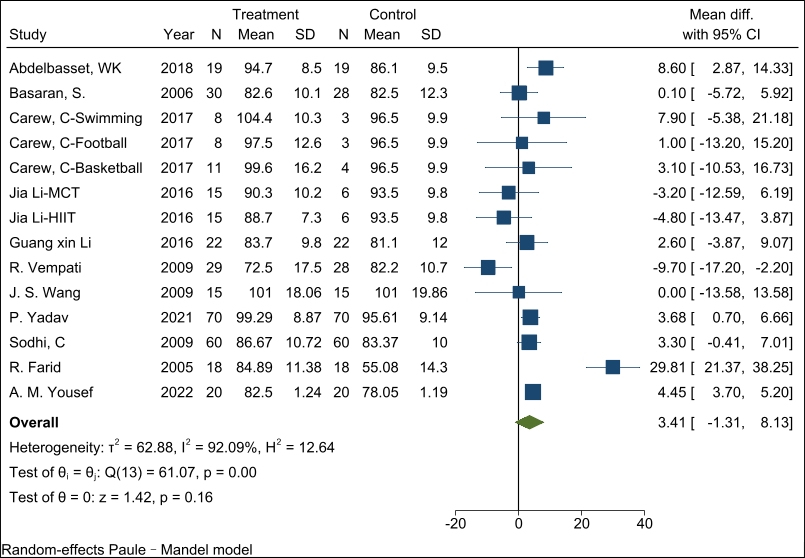


**Supplementary Figure 15.** Forest plot of the meta-analysis of physical activity on FVC% predicted using the Paule-Mandel (PM) heterogeneity variance estimator, performed as a sensitivity analysis. Effect estimates are expressed as mean differences with 95% confidence intervals. Mean diff, mean difference; SD, standard deviation; 95% CI, 95% confidence interval; FVC% predicted, forced vital capacity as a percentage of predicted value; PM, Paule-Mandel.
